# The Sno Oncogene Antagonizes Wingless Signaling during Wing Development in *Drosophila*

**DOI:** 10.1371/journal.pone.0011619

**Published:** 2010-07-16

**Authors:** Janine C. Quijano, Michael J. Stinchfield, Brad Zerlanko, Ying Y. Gibbens, Norma T. Takaesu, Cathy Hyman-Walsh, David Wotton, Stuart J. Newfeld

**Affiliations:** 1 School of Life Sciences, Arizona State University, Tempe, Arizona, United States of America; 2 Department of Biochemistry and Molecular Genetics, and Center for Cell Signaling, University of Virginia, Charlottesville, Virginia, United States of America; 3 Department of Genetics, Cell Biology and Development, University of Minnesota, Minneapolis, Minnesota, United States of America; University of Texas MD Anderson Cancer Center, United States of America

## Abstract

The Sno oncogene (Snoo or dSno in Drosophila) is a highly conserved protein and a well-established antagonist of Transforming Growth Factor-β signaling in overexpression assays. However, analyses of *Sno* mutants in flies and mice have proven enigmatic in revealing developmental roles for Sno proteins. Thus, to identify developmental roles for dSno we first reconciled conflicting data on the lethality of *dSno* mutations. Then we conducted analyses of wing development in dSno loss of function genotypes. These studies revealed ectopic margin bristles and ectopic campaniform sensilla in the anterior compartment of the wing blade suggesting that dSno functions to antagonize Wingless (Wg) signaling. A subsequent series of gain of function analyses yielded the opposite phenotype (loss of bristles and sensilla) and further suggested that dSno antagonizes Wg signal transduction in target cells. To date Sno family proteins have not been reported to influence the Wg pathway during development in any species. Overall our data suggest that dSno functions as a tissue-specific component of the Wg signaling pathway with modest antagonistic activity under normal conditions but capable of blocking significant levels of extraneous Wg, a role that may be conserved in vertebrates.

## Introduction

Transforming Growth Factor-β (TGF-β) family members perform essential tasks during development in all animals more complex than sponges [1]. Later in life, mutations that disrupt TGF-β signaling pathways upset homeostasis and in humans this can lead to tumors. In large measure, TGF-β functions are implemented in target cells by Smad tumor suppressor genes that function as signal transducers and transcription factors [2]. Analyses of Smads have identified many proteins that regulate their activity. Among the Smad regulators are oncogenic Sno family proteins that bind to Smad4.

The vertebrate Sno (*ski*-related novel gene) protein shares significant amino acid identity with the viral oncogene *v-ski* and Sno overexpression causes transformation of chick embryo fibroblasts. Sno is present as a single copy in the human genome but multiple promoters and alternative splicing generate six distinct transcripts. Four isoforms of the Sno protein have been identified with the longest isoform known as SnoN. In cancer, high levels of SnoN are correlated with poor outcome in estrogen-receptor positive breast tumors and gene amplification at the *Sno* locus is associated with squamous cell carcinoma of the esophagus. Mechanistic studies in mammalian cells revealed that SnoN, as part of a histone deacetylase complex, binds to Smad4 and blocks its ability to transduce TGF-β signals. As a result, Sno proteins were initially thought to be obligate antagonists of TGF-β signaling [3].

Our analysis in Drosophila suggested that Sno (formally Snoo in Flybase but most commonly referred to as dSno) has a subtler role in TGF-β signaling - as a pathway switch. We found that overexpression of dSno resulted in small wings with multiple vein truncations suggesting antagonism for TGF-β family members in the Decapentaplegic/Bone Morphogenetic Protein (Dpp/BMP) subfamily. Alternatively, *dSno* mutants displayed optic lobe defects in the larval brain similar to those present in *baboon* and *dSmad2* mutants suggesting a positive role in Activin signaling (Activin belongs to the other major subfamily of TGF-β proteins). Biochemical studies revealed that Medea - dSno complexes have reduced affinity for Mad and increased affinity for dSmad2 such that in the presence of dSno, Activin signaling is stimulated and Dpp signaling is reduced. The possibility that Sno proteins function as pathway switches in mammals is supported by data that SnoN facilitates Activin signaling in lung epithelial cells and cerebellar neurons [4], [5].

Surprisingly, studies of *Sno* mutants in both flies and mice have proven enigmatic in revealing developmental roles for Sno proteins, particularly with regard to any requirement for viability. One study of *SnoN* knockout mice reports early embryonic lethality for homozygous mutant embryos [6]. A second study reports that homozygous *SnoN* mutants are viable and that these mice have a defect in T-cell activation [7]. In 2006 we reported that *dSno* mutations are homozygous lethal at the larval/pupal transition and that the lethality is rescued to adulthood by expression of UAS.dSno [8]. Subsequently, three groups reported that individuals homozygous for *dSno* mutations could survive to adulthood [9]–[11]. Alternatively, all four groups reported identical results (Dpp antagonism) with independently derived UAS.dSno constructs.

To gain insight into dSno's role in development we first reconciled the conflicting data on the lethality of *dSno* mutants. Then we conducted loss of function studies utilizing *dSno* mutants and mutant clones paired with gain of function experiments employing Gal4 driven UAS.dSno. When these paired experiments generated complementary results it increased our confidence that the phenotypes revealed a true role for dSno. We found that *dSno* restricts Wingless (Wg) signaling in wing imaginal disks. Further we found that dSno accomplishes this by antagonizing Wg signal transduction in target cells. Overall our data suggest that dSno functions as a tissue-specific protein in Wg signaling with modest inhibiting activity under normal conditions but that can effectively block ectopic Wg signals.

## Results

### *dSno* mutant clones display ectopic expression of a Wg target gene in wing disks

Numerous studies have found that overexpression of dSno results in small wings with multiple vein truncations suggesting that dSno is capable of blocking Dpp/BMP subfamily signaling [8]–[11]. However, as *dSno* is broadly expressed in the wing pouch when compared to the narrow stripe of *dpp* expression [8] we wondered if opposition to Dpp signaling was dSno's true role in wing development. If this is the case, then a prediction of the “Dpp antagonism” hypothesis is that *dSno* mutant clones would result in Dpp overexpression phenotypes such as those seen with UAS.Mad or UAS.Medea - ectopic veins and enlarged wings.

Prior to initiating studies of somatic clones we further characterized the homozygous lethal *dSno* excision mutants *dSno^Ex17B^* and *dSno^Ex4B^* (Text S1). DNA sequencing (Figure S1) and RNA in situ hybridization (Figure S2) revealed that *dSno^Ex17B^* is a strong hypomorph and *dSno^Ex4B^* is a protein null. We also performed complementation and stage of lethality tests (Figure S3) with *dSno^174^* - a deletion of most of the dSno protein that is reported as homozygous viable at nearly 50% of wild type levels [11]. Taken together the *dSno^174^* studies suggest that: 1) all of the reported *dSno* mutants are likely allelic, 2) the extent of viability for *dSno* homozygous deletions varies between laboratories due to environmental factors, and 3) a developmental role for *dSno* is to facilitate Activin signaling during optic lobe development as we reported previously [8].

To test the “Dpp antagonism” hypothesis, we first conducted preliminary experiments employing unmarked clones of cells homozygous for *dSno^Ex4B^*, *dSno^Ex17B^* or *dSno^sh1402^* in adult wings (Text S1). Wings with unmarked clones for any allele displayed ectopic margin bristles on the wing blade (Figure S4B). Though restricted to distal regions of the anterior compartment, the phenotype is similar to the ectopic bristle phenotype generated by loss of Wg antagonism in *zeste white3* mutant clones (*zw3^M11^*) [12], [13] or by ectopic Wg signaling via expression of UAS.Dishevelled (Dsh) [14]. We then inspected the wings of *dSno^174^* homozygous escapers and found they display ectopic margin bristles in the anterior compartment (n = 18; Figure S4E) and ectopic campaniform sensilla on wing vein L3 (Figure S5C). Wings from another *dSno* mutant allele *dSno^GS-c517^* [10] when in trans to *dSno^Ex4B^* also exhibit ectopic margin bristles in the anterior compartment and ectopic sensilla (n = 136; Figure S4F). Reexamination of wings with *zeste white3* mutant clones revealed ectopic sensilla on the L3 vein (Figure S5D). The presence of ectopic bristles and sensilla in three independently derived *dSno* mutants indicates that they result from the loss of *dSno*.

The similarity of the wing phenotypes for *dSno* and *zw3* mutants suggests the hypothesis that they both function as antagonists of Wg signaling. In canonical Wg signal transduction the dFrizzled2 receptor activates Dsh, which then relays the signal to a cytoplasmic protein complex. This complex includes the antagonists Zw3, dAPC1/dAPC2, dAxin and the positively acting Armadillo (Arm). Under nonsignaling conditions Zw3 phosphorylates Arm tagging it for destruction. Upon receipt of a Wg signal Arm is released from the complex, enters the nucleus and partners with transcription factors (e.g., dTCF or Pygopus) to activate gene expression [15]–[17]. Among its roles, Wg regulates the formation of sensilla and margin bristles in the wing [18].

**Figure 1:**
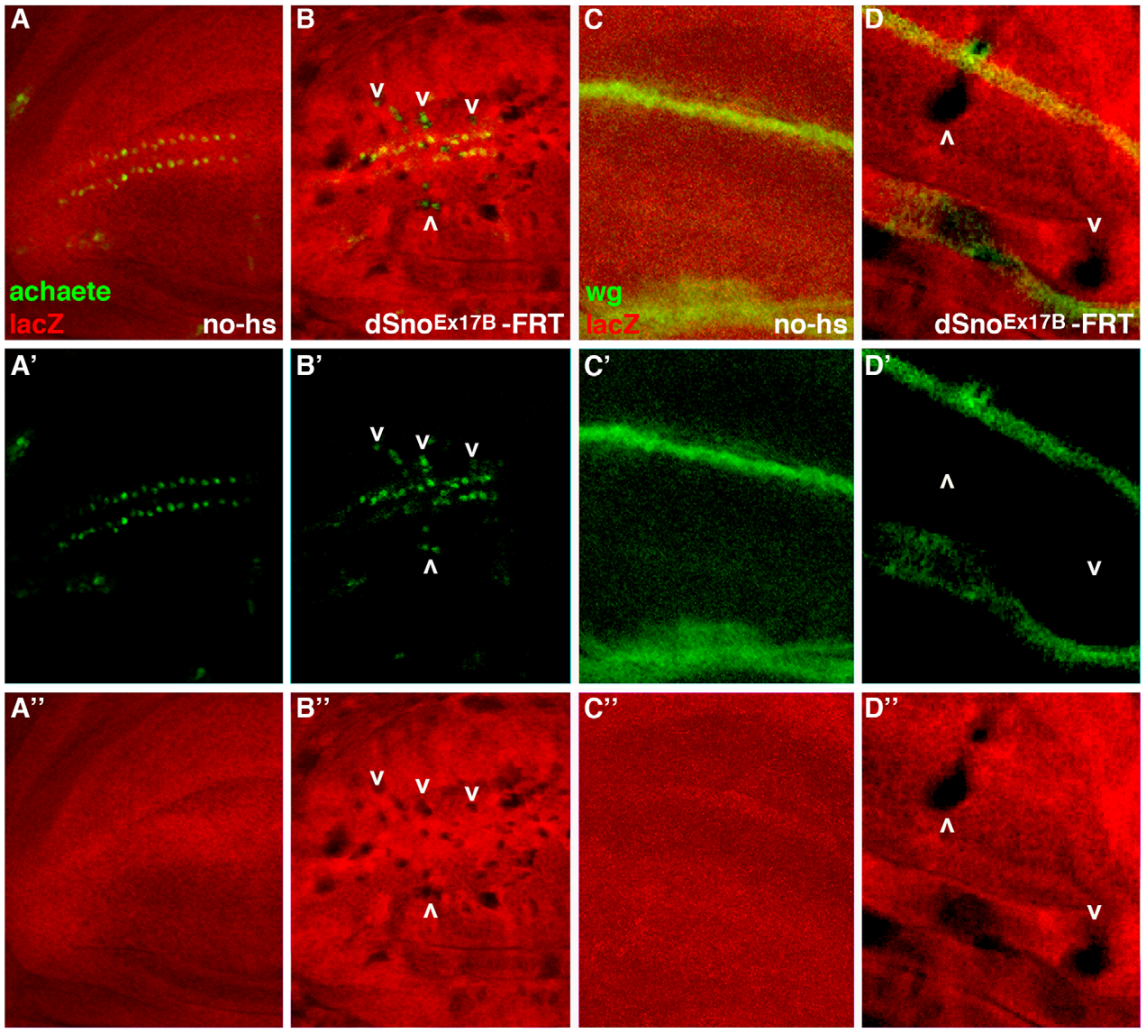
*dSno* clones in the wing generate ectopic expression of a Wg target gene but do not affect Wg expression. *dSno^Ex17B^* FRT40A/Arm-lacZ FRT40A third instar wing disk with a focus on the wing pouch and anterior margin primordia. (A, A', A”) Disk without heat shock stained with anti-Ac (green) and anti-lacZ (red) shown merged and as individual channels. Arm-lacZ is ubiquitously expressed. (B, B', B”) Disk with hs-FLP-induced *dSno* mutant clones. Clones of cells homozygous for *dSno^Ex17B^* are seen via the absence of lacZ. Loss of *dSno* does not affect normal nuclear Ac expression and numerous mutant clones outside this area within the anterior compartment display ectopic Ac expression (arrowheads). (C, C', C”) Disk without heat shock stained with anti-Wg (green) and anti-lacZ (red). (D, D', D”) Disk with hs-FLP-induced *dSno* mutant clones. Loss of *dSno* does not affect normal Wg expression and mutant clones outside this area, in either the anterior or posterior compartment, do not display ectopic Wg (arrowheads). Clones at the anterior-posterior compartment boundary that encompass both cell layers and bisect the Wg stripe appear to support increased Wg diffusion into the ventral but not the dorsal compartment (n = 6).

To molecularly test this hypothesis we generated marked clones for *dSno^Ex17B^* or *dSno^sh1402^* in third instar larval wing disks.Results with both alleles were consistent and those of *dSno^Ex17B^* are shown. We examined the expression of Achaete (Ac), a target of Wg signaling in sensory organ precursor cells that will become bristles on the dorsal and ventral surfaces of the anterior wing margin [19]. Our *dSno* RNA in situ data [8] indicated that Ac expression is completely encompassed by *dSno* expression. We found that *dSno* clones do not affect normal Ac expression but they generate ectopic Ac on the presumptive wing blade (Figure 1B) in the anterior compartment. Note that Ac expression is restricted to the anterior compartment by a mechanism that is independent of Wg [20] and thus *dSno* clones in the posterior compartment do not express ectopic Ac.

To eliminate the possibility that ectopic Ac resulted from alterations in Wg expression we then stained wing disks bearing marked *dSno* mutant clones with an antibody to Wg (Figure 1D). This experiment shows that the loss of *dSno* does not affect normal Wg expression from the presumptive margin and that mutant clones outside this area do not display ectopic Wg (though clones at the anterior-posterior compartment boundary appear to support increased Wg diffusion into the ventral compartment). We conclude that dSno does not regulate Wg expression nor the expression of Zw3 (data not shown) and that the effect of *dSno* mutant clones on Ac is due to a role in restricting Wg signal transduction.

### dSno rescues lethality due to overexpression of Wg but not Notch pathway components

Our first gain of function experiment was designed to determine if dSno was capable of sufficient antagonism for Wg signaling to overcome overexpression of the Wg pathway signal transducer Dsh. For these analyses we employed the wing-specific MS1096.Gal4, a homozygous viable insertion in the second intron of the Beadex gene on the X chromosome. Evidence that MS1096.Gal4 is exclusive to the wing derives from the two reports: complete deletion of the Beadex locus results only in wing defects [21] and crosses to UAS.lacZ show meaningful staining only in the wing imaginal disk [22].

**Figure 2:**
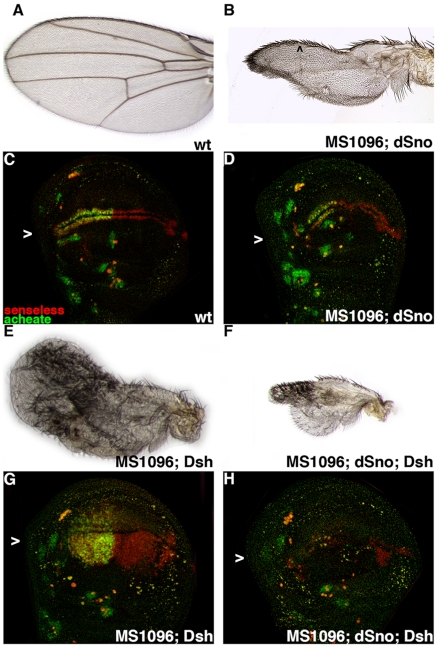
dSno rescues overexpression of Dsh in the wing. (A) Wild type wing. (B) MS1096.Gal4; UAS.dSno wing is small, has gaps in the row of wide-spaced chemosensory bristles on the dorsal surface of the wing margin (arrowhead), no L3 sensilla or veins on the wing blade. (C) Wild type disk labeled for Ac (green) and Sens (red). Expression of both proteins in two rows of cells adjacent to the wing margin that will become bristles in the adult wing is visible with Ac present only in cells of the anterior compartment (arrowhead). (D) MS1096.Gal4; UAS.dSno disk has reduced Ac and Sens expression along the presumptive wing margin (arrowhead) and in the center of the disk below the margin stripe corresponding to L3 sensilla precursors. (E) MS1096.Gal4; UAS.Dsh wing is large, has no adhesion between the dorsal and ventral surfaces, no veins or obvious margin and numerous ectopic bristles on both surfaces of the wing blade. (F) MS1096.Gal4; UAS.dSno, UAS.Dsh wing is small, has no veins and a greatly reduced number of ectopic bristles on the wing blade. (G) MS1096.Gal4; UAS.Dsh disk has extensive ectopic expression of Ac and Sens though Ac is limited to the anterior compartment. (H) MS1096.Gal4; UAS.dSno, UAS.Dsh disk has reduced Ac and Sens expression even when compared to wild type.

MS1096.Gal4 expression of UAS.dSno does not affect viability (51 experimental flies compared to 50 siblings). These flies have small and veinless wings (n = 102), as expected due to antagonism of Dpp signaling (Figure 2B). These wings have no sensilla on the L3 vein and gaps in the row of wide-spaced chemosensory bristles along the anterior margin. The loss of margin bristles is also seen when Wg signal transduction is compromised in *arm* mutant clones (*arm^4^*; Figure S4D). The similarity of the phenotypes generated by dSno overexpression and *arm* loss of function again suggest that a role for dSno is to antagonize Wg signaling, consistent with the similarity of *dSno* and *zw3* loss of function data. We confirmed that the loss of the L3 sensilla in dSno expressing wings was not due to Dpp antagonism in assays with Scabrous.Gal4 driving UAS.dSno or UAS.Mad-RNAi. In these experiments UAS.dSno expression eliminated the L3 vein and the L3 sensilla while UAS.Mad-RNAi expression eliminated the L3 vein but not the L3 sensilla (Figure S5F and 5G).

When we expressed UAS.Dsh with MS1096.Gal4 we found near-absolute lethality (11 experimental flies were obtained from 1298 pupae - an eclosion rate of 0.84%). The wings of rare escapers lack surface adhesion, veins and an obvious wing margin. Instead they display a “lawn” of ectopic bristles on both wing surfaces. (Figure 2E). In this genotype, careful observation revealed that lethality occurred at the pharate stage when ectopic bristles, particularly those on the dorsal side, trapped the individual within the pupal case and prevented them from eclosing.

Coexpression of UAS.dSno and UAS.Dsh with MS1096.Gal4 resulted in nearly complete rescue of lethality with 90.8% of the expected experimental flies observed (n = 564). The wings (n = 40) of rescued flies are smaller than UAS.dSno wings and also have no veins (Figure 2F). The number of ectopic bristles is significantly suppressed on the UAS.dSno and UAS.Dsh wings when compared to wings expressing UAS.Dsh alone, suggesting a basis for the rescue of lethality. The hypothesis is that in the coexpressing wing a sufficient amount of excess Wg signaling, engendered by Dsh overexpression, has been antagonized by dSno such that these individuals can now eclose. We briefly entertained the alternative hypothesis that the reduction in wing size generated by coexpressing UAS.dSno, an additive effect rather than Wg antagonism, was responsible for rescue of UAS.Dsh lethality. However, the alternative does not explain the reduction in the number of ectopic bristles on the wings of rescued flies We eliminated a second alternative hypothesis, that these results are specific to MS1096.Gal4, by reproducing the rescue of UAS.Dsh wing phenotypes by UAS.dSno coexpression with Scabrous.Gal4 (n = 538; Figure S5H and 5I).

We then tested the Wg antagonism hypothesis molecularly by examining gene expression in third instar wing disks. MS1096.Gal4 expression of UAS.dSno led to a modest reduction in the expression in disks (n = 7; Figure 2D) of two Wg target genes found in sensory organ precursor cells, Ac and Senseless (Sens). Alternatively UAS.Dsh overexpression led to widespread ectopic expression of these genes in disks (n = 4; Figure 2G), consistent with the presence of numerous ectopic bristles in wings of this genotype. Ectopic expression of Ac and Sens was strongly suppressed when dSno was coexpressed with Dsh (n = 5; Figure 2H). Coexpression of the Wg antagonist dAxin also fully suppressed mutant phenotypes due to the overexpression of Dsh [14]. Together these results suggest that dSno antagonizes Wg signal transduction downstream of Dsh.

However Dsh has been reported to function as a positive factor in the Wg pathway and as a negative factor in Notch signaling in wing disks where Notch is also required for margin bristle development [23]. Thus, to rule out a role for the Notch pathway in UAS.dSno rescue of UAS.Dsh phenotypes we conducted a parallel experiment with a constitutively active form of Notch (CA-Notch). Expression of UAS.CA-Notch with MS1096.Gal4 leads to absolute lethality (no adults from 1809 pupae) and this does not change when dSno is coexpressed (no adults from 1867 pupae).

**Figure 3:**
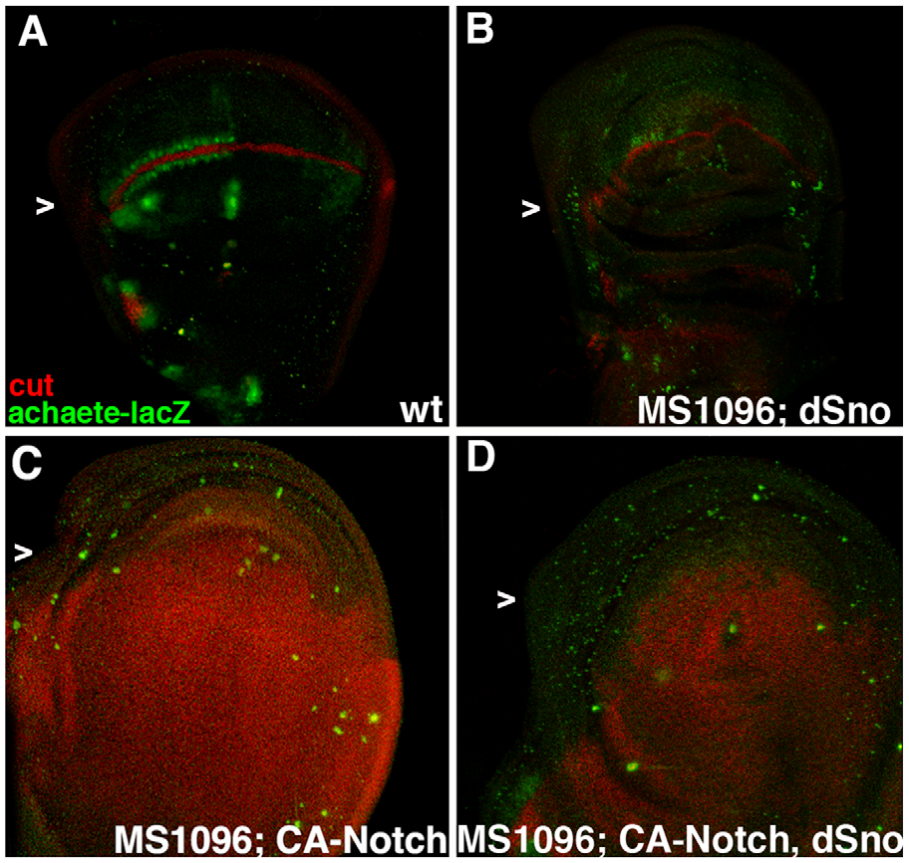
dSno cannot rescue constitutively active Notch. (A) MS1096.Gal4; Ac-lacZ disk labeled with anti-lacZ to display Ac expression (green) and anti-Cut (red). Expression in three rows of cells either adjacent to (Ac) or on (Cut) the wing margin that will become bristles in the adult wing is visible with Ac only present in cells of the anterior compartment (arrowhead). (B) MS1096.Gal4; UAS.dSno, Ac-lacZ disk has reduced Ac-lacZ expression but largely normal Cut expression. (C) MS1096.Gal4; UAS.CA-Notch, Ac-lacZ disk has no Ac-lacZ expression but nearly ubiquitous expression of Cut. (D) MS1096.Gal4; UAS.dSno, UAS.CA-Notch, Ac-lacZ disk is qualitatively the same as UAS.CA-Notch alone.

We then examined the expression of Ac (Wg target) and Cut (Notch target) [24] in sensory organ precursor cells in wing disks. UAS.dSno generates disks with reduced Ac expression but normal Cut expression suggesting that UAS.dSno does not influence this Notch pathway target (n = 7; Figure 3B). The CA-Notch lethal genotype generates disks that are much larger than wild type, have nearly ubiquitous expression of Cut and essentially no Ac expression (Figure 3C). The widespread expression of the sensory organ precursor cell marker Cut in these disks is reminiscent of the widespread expression of Ac and Sens in UAS.Dsh disks that lead to ectopic bristles in adults (compare Figure 3C with 2G). UAS.dSno and UAS.CA-Notch disks (n = 7; Figure 3D) reveal no influence of UAS.dSno as they appear essentially the same as those expressing UAS.CA-Notch alone. This contrasts with disks coexpressing UAS.dSno and UAS.Dsh in which the widespread expression of Ac and Sens is largely suppressed (compare Figure 3D with 2H). These results suggest that UAS.dSno rescue of UAS.Dsh phenotypes is not due to effects on CA-Notch signaling.

**Figure 4:**
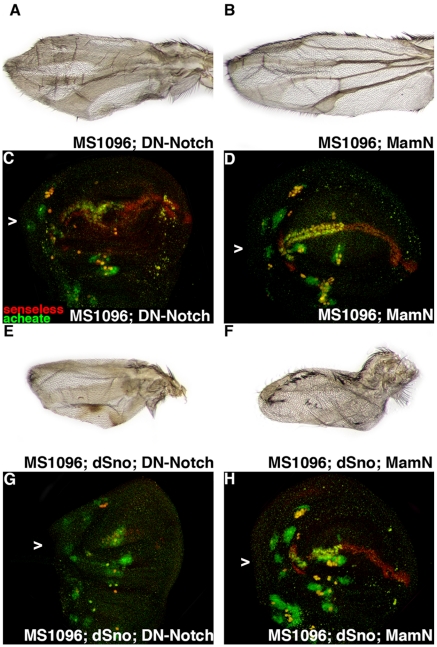
dSno cannot rescue dominant negative Notch or Mastermind. (A) MS1096.Gal4; UAS.DN-Notch wing is small, has no veins and very few anterior margin bristles. (B) MS1096.Gal4; UAS.MamN wing is smaller than wild type but modestly larger than the UAS.DN-Notch wing, has vein defects and gaps in the anterior margin bristles. (C) MS1096.Gal4; UAS.DN-Notch disk labeled with anti-Ac (green) and anti-Sens (red). Expression in cells adjacent to the wing margin is indicated (arrowhead). The disk has reduced Ac and Sens expression compared to the wild type disk in Fig. 2C. (D) MS1096.Gal4; UAS.MamN disk has approximately wild type Ac and Sens expression. (E) MS1096.Gal4; UAS.dSno, UAS.DN-Notch wing is smaller than UAS.DN-Notch alone, is veinless and has no margin bristles. (F) MS1096.Gal4; UAS.dSno, UAS.MamN wing is smaller than UAS.MamN alone, is veinless and the anterior margin bristles are completely disorganized (G) MS1096.Gal4; UAS.dSno, UAS.N-Notch disk has less Ac and Sens expression than UAS.DN-Notch alone. (H) MS1096.Gal4; UAS.dSno, UAS.MamN disk has less Sens and Ac expression then UAS.MamN alone.

To be certain that dSno does not play any role in Notch signaling in wing development we conducted coexpression experiments with dominant negative forms of both Notch (UAS.DN-Notch) and the Notch pathway transcription factor Mastermind (UAS.MamN). When expressed with MS1096.Gal4, UAS.DN-Notch leads to significant lethality with 12% of the expected experimental flies observed (93 experimental compared to 659 siblings). These wings (n = 34) are small, have no veins and very few anterior margin bristles (Figure 4A). UAS.MamN expression modestly reduces Notch signaling and does not cause lethality with 95.8% of the expected flies observed (595 experimental compared to 648 siblings). Wings (n = 684) of this genotype are smaller than wild type but larger than UAS.DN-Notch wings, they have veins with distal truncations and there are gaps in the anterior margin bristles (Figure 4B).

Coexpressing UAS.dSno and UAS.DN-Notch generates additional lethality with only 4.0% of expected adults observed (34 experimental compared to 812 siblings). These wings (n = 45) display additive effects of each gene's overexpression. Coexpressing wings are smaller than either parental wing, veinless and have lost all their margin bristles (Figure 4E). Coexpressing UAS.dSno and UAS.MamN generates a low level of lethality with 89.2% of expected adults observed (235 experimental compared to 292 siblings). These wings (n = 45) also display additive effects. Coexpressing wings are smaller than UAS.MamN wings, have no veins and the anterior margin bristle rows are completely disorganized (Figure 4F).

An examination of wing disks also indicates that dSno coexpression does not rescue but rather exacerbates phenotypes due to UAS.DN-Notch and UAS.MamN. UAS.DN-Notch expressing disks have little Ac or Sens expression (n = 7; Figure 4C). UAS.dSno, UAS.DN-Notch coexpressing disks have lost Ac and Sens expression (n = 3; Figure 4G). UAS.MamN expressing disks display roughly wild type Ac and Sens expression (n = 8; Figure 4D). UAS.dSno, UAS.MamN coexpressing disks contain reduced Ac and Sens expression (n = 8; Figure 4H). Results from these dominant negative Notch pathway experiments argue against interactions between dSno and DN-Notch signaling.

We also examined the expression of antibodies to the Notch intracellular domain and to the Notch ligands Delta and Serrate in wing disks with *dSno^EX17B^* and *dSno^sh1402^* mutant clones. This analysis showed that *dSno* clones have no effect on Notch, Delta or Serrate expression (data not shown). Taken together the negative results from our examination of interactions between dSno and the Notch pathway lend support to the hypothesis that a normal role for dSno is the restriction of Wg signal transduction during wing development.

### dSno is fully epistatic to Zw3 and dAxin but partially epistatic to Arm in the Wg pathway

**Figure 5:**
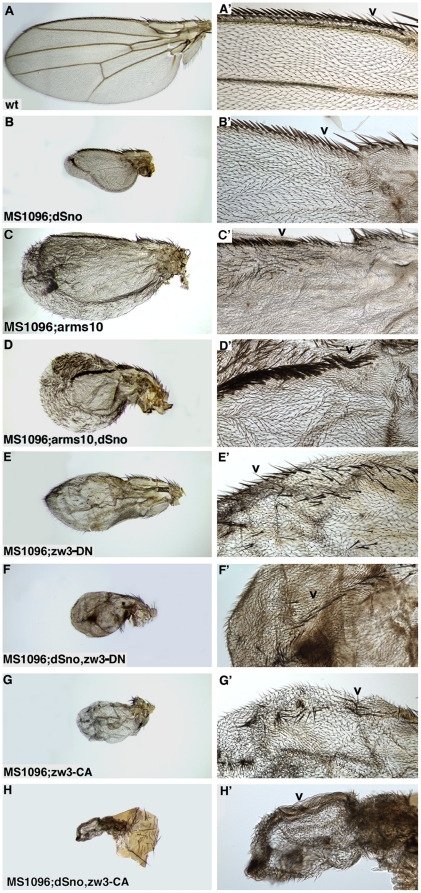
dSno is epistatic to Zw3 but not Arm in wing margin bristle development. Dorsal views of adult wings. High magnification focused on triple row region of anterior margin bristles that develop from Ac and Sens expressing cells (arrowhead). (A, A') Wild type wing with tightly spaced stout mechanosensory bristles atop the margin and widely spaced chemosensory bristles on the dorsal surface. (B, B') MS1096.Gal4; UAS.dSno wing is small and has no veins. The row of stout mechanosensory bristles appears wild type but the row of chemosensory bristles is irregularly spaced. (C, C') MS1096.Gal4; UAS.Arm^S10^ wing with strong ectopic Wg signaling lacks adhesion between the dorsal and ventral surfaces, has no veins and there are numerous ectopic bristles on both surfaces. The margin displays multiple rows of tightly spaced, stout mechanosensory bristles with all other bristle types missing. (D, D') MS1096.Gal4; UAS.Arm^S10^, UAS.dSno wing is smaller than the UAS.Arm^S10^ wing and has no ectopic bristles on its dorsal surface. Ectopic bristles remain on the ventral surface and the margin displays multiple rows of tightly spaced, stout mechanosensory bristles with all other bristle types missing. (E, E') MS1096.Gal4; UAS.Zw3-DN wing expressing dominant negative Zw3 has modest ectopic Wg signaling. The wing is smaller than wild type, has no veins and there are ectopic margin bristles on the dorsal and ventral surfaces. All bristle types appear to be present on the margin but individual rows are difficult to identify. (F, F') MS1096.Gal4; UAS.dSno, UAS.Zw3-DN wing is small and has no veins. There is now a distinct row of margin bristles though its content is mixed and only a few ectopic bristles remain on the wing blade. (G, G') MS1096.Gal4; UAS.Zw3-CA wing expressing constitutively active Zw3 has reduced Wg signaling. The wing is smaller than wild type and has no veins. The row of stout mechanosensory bristles is sparse compared to wild type and there are no ectopic margin bristles. (H, H') MS1096.Gal4; UAS.dSno, UAS.Zw3-CA wing is smaller than either UAS.dSno or UAS.Zw3-CA alone and has no veins or margin bristles.

At this point our data suggests that dSno operates at or between Dsh and the target gene Ac in the Wg pathway. To further clarify where in the Wg pathway dSno functions we conducted additional coexpression experiments. We began with a constitutively active form of Arm, Arm^S10^ [25]. Expression of UAS.Arm^S10^ with MS1096.Gal4 is not quite as lethal as UAS.Dsh - 4.6% of the expected number of adults was observed (26 experimental compared to 1087 siblings). These wings bear the hallmarks of ectopic Wg signaling. UAS.Arm^S10^ wings (n = 52) lack surface adhesion, are veinless and display numerous ectopic margin bristles on both the dorsal and ventral surfaces (Figure 5C). In contrast to the marginless UAS.Dsh wing, the UAS.Arm^S10^ wings retain a distinct margin but the anterior region is composed of multiple rows of tightly spaced stout mechanosensory bristles with other types of bristles absent.

When UAS.dSno is coexpressed with UAS.Arm^S10^ there is modestly improved survival with 23% of the expected UAS.dSno, UAS.Arm^S10^ flies observed (131 experimental compared to 1005 siblings). The surviving UAS.dSno, UAS.Arm^S10^ flies (n = 262) display similarities and differences from UAS.Arm^S10^ wings. Wings from UAS.dSno, UAS.Arm^S10^ flies are smaller and the ectopic bristle phenotype is completely suppressed on the dorsal surface. However, they still display ectopic bristles on the ventral surface and multiple rows of tightly packed stout mechanosensory bristles on the anterior margin with other rows of bristles absent (Figure 5D). These mixed epistasis results, partial rescue of some aspects of the phenotype but failure to suppress others suggest that dSno antagonism of Wg signal transduction occurs at the level of Arm or above. As noted previously, an additive effect of dSno rather than Wg antagonism might explain the increase in viability of UAS.Arm^S10^ and UAS.dSno coexpressing flies but it does not explain the reduction in the number of ectopic bristles on their wings.

We then examined wings coexpressing dSno and a dominant negative form of Zw3 (Zw3-DN has an A81T mutation in an invariant alanine within the kinase domain) or a constitutively active form of Zw3 (Zw3-CA has an S9A mutation in an inhibiting phospho-serine) [26]. When expressed with MS1096.Gal4, UAS.Zw3-DN results in modest overactivation of Wg signaling with 66.6% of the expected number of adults observed (27 experimental compared to 54 siblings). Adults of this genotype have wings (n = 22) that are smaller than wild type, lack surface adhesion and are veinless. There are ectopic margin bristles on the dorsal and ventral surface (Figure 5E) but far fewer than for UAS.Arm^S10^. While all bristle types appear to be present on the margin, specific rows are difficult to identify. Coexpression of UAS.dSno, UAS.Zw3-DN resulted in full rescue of lethality (351 experimental compared to 295 siblings) and suppression of the ectopic bristle phenotype. Further the coexpressing UAS.dSno, UAS.Zw3-DN wings (n = 40) now display a distinct row of margin bristles though its content is mixed (Figure 5F). The rescue of lethality as well as the suppression of the ectopic bristle and margin phenotypes suggests that dSno antagonism of Wg signaling occurs at or below Zw3.

Alternatively, MS1096.Gal4 driven UAS.Zw3-CA results in modestly reduced Wg signaling with little lethality - 91% of the expected number of adults was observed (42 experimental compared to 51 siblings). Adults of this genotype have wings (n = 24) that are smaller than wild type and have no veins. The row of stout mechanosensory bristles on the margin is sparse compared to wild type and there are no ectopic margin bristles (Figure 5G). The UAS.dSno, UAS.Zw3-CA coexpressing genotype shows no lethality (285 experimental compared to 203 siblings). UAS.dSno, UAS.Zw3-CA wings (n = 40) are smaller than either UAS.dSno or UAS.Zw3-CA alone and have no veins. However, the coexpressing wings also have no anterior margin bristles (Figure 5H) even though in UAS.dSno wings the anterior margin has only minor defects (Figure 5B). The enhancing effect of UAS.dSno on the UAS.Zw3-CA margin bristle phenotype suggests dSno and Zw3 both have negative effects on Wg signal transduction and that dSno impacts Wg signaling at or below Zw3.

**Figure 6:**
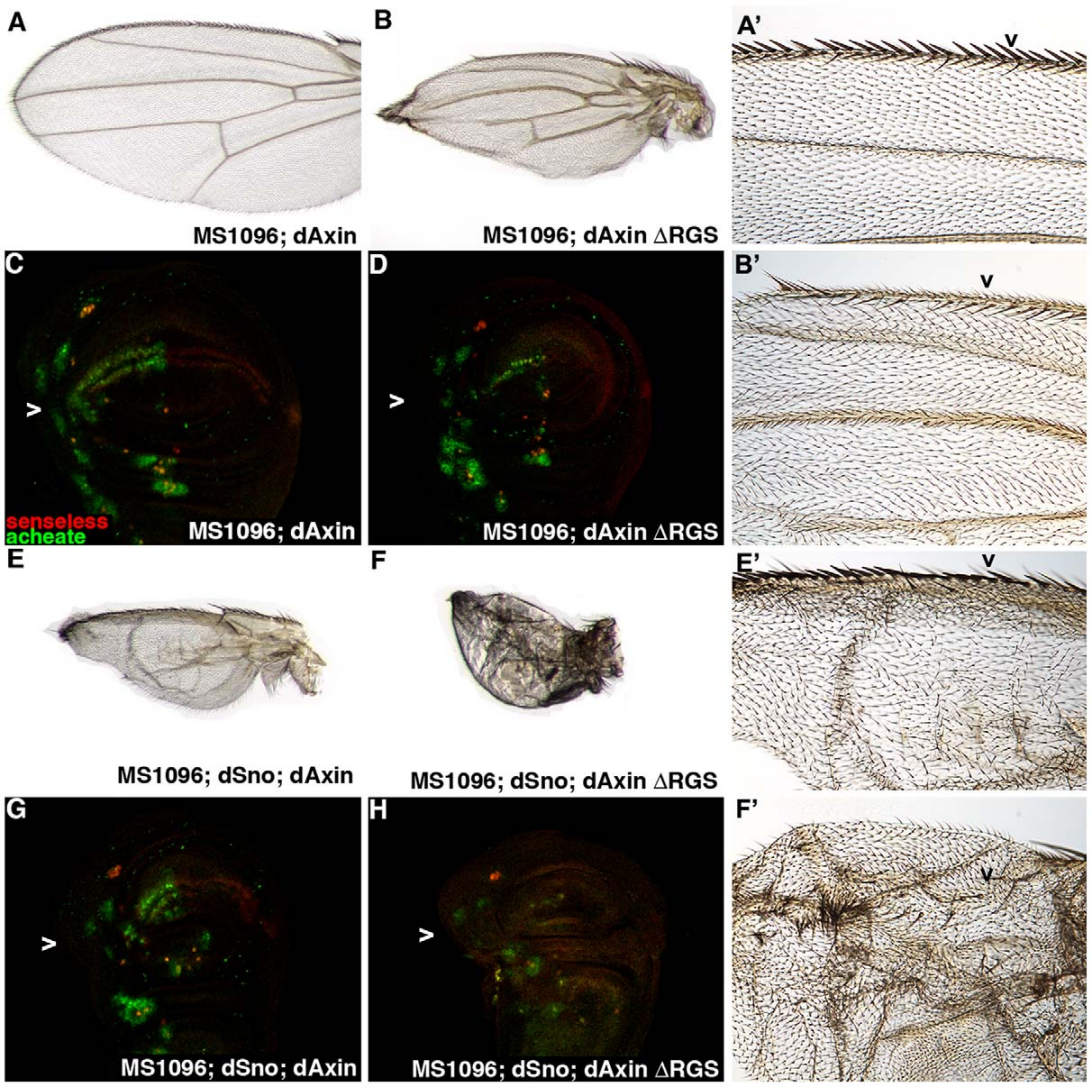
dSno is epistatic to dAxin in wing development. Adult wings. High magnification focused on triple row region of anterior margin bristles that develop from Ac and Sens expressing cells (arrowhead). (A,A') MS1096.Gal4; UAS.dAxin wing appears wild type. (B, B') MS1096.Gal4; UAS.dAxin ΔRGS (the deletion confers modest constitutive activity resulting reduced Wg signaling) is smaller and narrower than wild type, has truncated longitudinal veins and truncated rows of anterior margin bristles. There is nearly complete loss of the row of stout mechanosensory bristles atop the margin but a largely normal row of alternating thin mechanosensory and chemosensory bristles is present on the ventral surface. (C) MS1096.Gal4; UAS.dAxin disk labeled with anti-Ac (green) and anti-Sens (red). Expression in cells adjacent to the wing margin is indicated (arrowhead). This disk appears wild type. (D) MS1096.Gal4; UAS.dAxinΔRGS disk has greatly reduced Ac expression and no Sens expression along the presumptive margin. (E, E') MS1096.Gal4; UAS.dSno, UAS.dAxin wing is smaller then, UAS.dSno or UAS.dAxin alone, veinless and there are disruptions in the row of widely spaced chemosensory bristles on the dorsal surface. (F, F') MS1096.Gal4; UAS.dSno, UAS.dAxinΔRGS wing is smaller than UAS.dSno or UAS.dAxinΔRGS alone, is veinless and has randomly scattered bristles on the anterior margin - similar to the UAS.Zw3-CA wing in 5G. (G) MS1096.Gal4; UAS.dSno, UAS.dAxin disk has interrupted and disorganized Ac and Sens expression. (H) MS1096.Gal4; UAS.dSno, UAS.dAxinΔRGS disk has very little Ac expression and no Sens.

Subsequently we analyzed wings coexpressing dSno and dAxin or dSno and dAxinΔRGS (dAxinΔRGS has a deletion of the dAPC-interacting RGS domain that confers weak constitutive activity that results in modestly reduced Wg signaling) [27]. Expression of UAS.dAxin with MS1096.Gal4 was not lethal (753 experimental compared to 701 siblings). For this genotype wings (n = 14) and wing disks (n = 8) appeared wild type (Figure 6A, 6A' and 6C), consistent with a previous report [28]. MS1096.Gal4 expression of UAS.dAxinΔRGS also was not lethal (315 experimental compared to 332 siblings). These wings (n = 13) display several features resulting from reduced Wg signaling (Figure 6B and 6B'). They are smaller and narrower than wild type with truncated longitudinal veins, truncated rows of anterior margin bristles and a nearly complete loss of the row of stout mechanosensory bristles atop the margin. MS1096.Gal4, UAS.dAxinΔRGS wing disks show reduced Ac expression and no Sens expression along the presumptive margin (n = 5; Figure 6D).

Coexpressing UAS.dSno and UAS.dAxin does not generate any lethality (49 experimental compared to 35 siblings) but the wing phenotype is enhanced (Figure 6E). These wings (n = 9) are more severely affected than either UAS.dSno alone or UAS.dAxin alone (compare Figure 6E with 2B and 6A). The wings are smaller, veinless and there are disruptions in the anterior margin bristle rows. In these disks Ac and Sens expression is interrupted and disorganized (n = 12; Figure 6G). MS1096.Gal4 driven UAS.dSno and UAS.dAxinΔRGS generates enhanced phenotypes as well. This genotype results in modest lethality (75.4%; 72 experimental compared to 119 siblings) when none is associated with either UAS.dSno alone or UAS.dAxin alone. Coexpressing wings (n = 8) are smaller than UAS.dSno alone or UAS.dAxinΔRGS alone (compare Figure 6F and F' with 2B and 6B), veinless and have only a few randomly scattered bristles on the anterior margin. These wings appear similar to Zw3-CA (compare Figure 6F and F' with Fig. 5G and G'). The coexpressing disks have very little Ac expression and no Sens (Figure n = 11; 6H). The enhancing effect of UAS.dSno expression on UAS.dAxin and UAS.dAxinΔRGS phenotypes is similar to that seen with UAS.dSno and UAS.Zw3-CA coexpression suggesting that all three proteins have negative effects on Wg signal transduction and that dSno impacts Wg signaling at or below dAxin.

### Brinker does not rescue overexpression of Dsh nor interact with dSno in the wing

dSno is not the first TGF-β antagonist to be implicated in inhibiting Wg signaling. The BMP antagonist Brinker (Brk) was previously shown to restrict Wg signaling in two embryonic tissues, the midgut and the ventral epidermis. Brk accomplishes this via repressor complexes containing Teashirt that compete for enhancer binding sites with Arm/dTCF activation complexes [29]. In addition, in follicle cell patterning during oogenesis dSno and Brk function together to antagonize Dpp signaling [11]. In studies designed to determine if *dSno* has any role during embryonic development preliminary data suggests that dSno blocks Wg signaling in the ventral epidermis (Figure S6). Thus, we examined the possibility that Brk antagonizes Wg during wing development and whether dSno might cooperate with Brk in this process.

**Figure 7:**
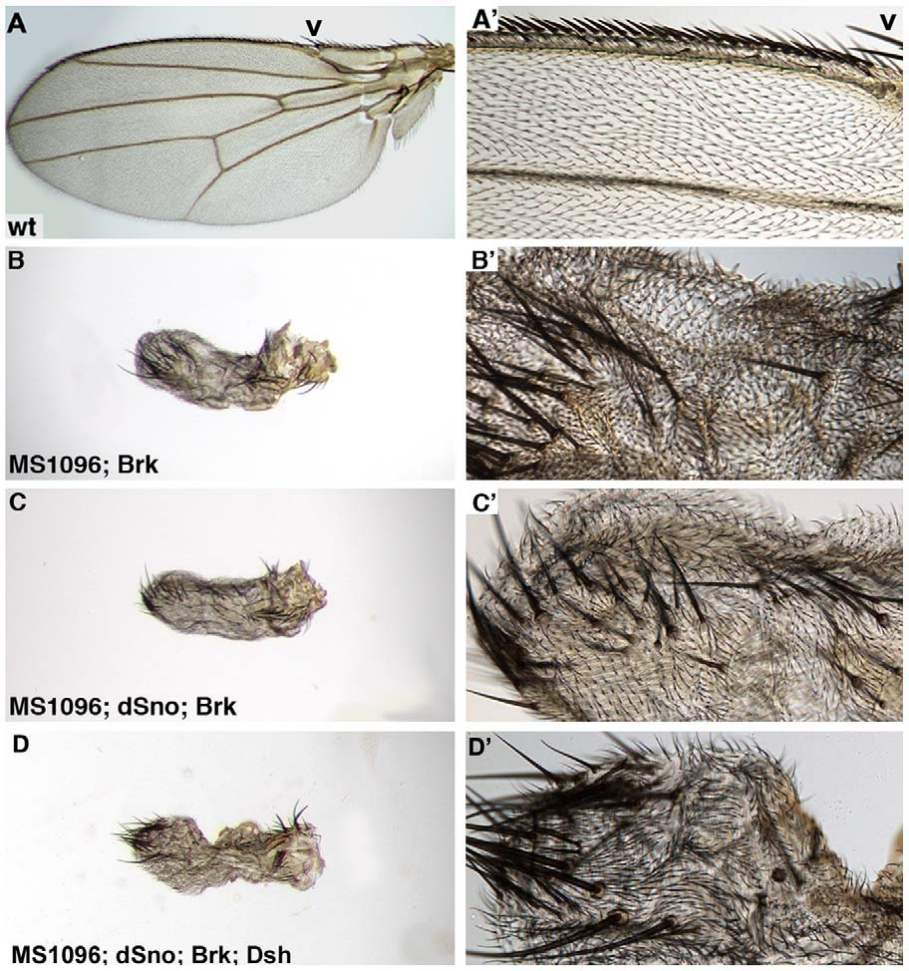
dSno does not interact with Brinker in the wing. Adult wings. High magnification focuses on the anterior margin bristles. (A, A') Wild type wing with tightly spaced stout mechanosensory bristles atop the margin and widely spaced chemosensory bristles on the dorsal surface. (B, B') MS1096.Gal4; UAS.Brk wing is small and has no veins. All normal margin bristle rows are absent and instead there is a disorganized row of ectopic bristles that appear similar to the pair of large bristles normally found at the distal tip of the costa (arrowhead). (C, C') MS1096.Gal4; UAS.dSno; UAS.Brk is similar to the UAS.Brk wing - no effect of dSno is evident. (D, D') MS1096.Gal4; UAS.dSno; UAS.Brk, UAS.Dsh is also similar to the UAS.Brk wing - again no effect of dSno is evident.

Expression of UAS.Brk with MS1096.Gal4 did not generate any lethality (866 experimental compared to 733 siblings). Adult wings (n = 40) were small, veinless and displayed a novel margin bristle phenotype. No normal margin bristles were evident but instead there were numerous ectopic bristles that appear similar to the pair of large bristles found normally on the margin at the distal tip of the costa (Figure 7A and 7B). Coexpression of UAS.Brk and UAS.Dsh with MS1096.Gal4 had no effect on the lethality engendered by overexpression of Dsh (0 experimental flies compared to 618 siblings). Coexpression of UAS.dSno with UAS.Brk did not generate any lethality (191 experimental compared to 134 siblings) and the presence of UAS.dSno had no effect on the UAS.Brk phenotype (Figure 7C). When UAS.Brk, UAS.dSno and UAS.Dsh were coexpressed there was complete rescue of UAS.Dsh generated lethality (206 experimental flies compared to 170 siblings) but the wings were identical to those expressing UAS.Brk alone (n = 38; Figure 7D). We conclude that Brk does not inhibit Wg signaling during wing development and therefore is not a partner for dSno as a Wg antagonist.

## Discussion

Molecular and genetic analyses of phenotypes generated in complementation tests with dSno alleles from three different laboratories reveal that they are alleles of the same gene. These studies also support our previous data that a developmental role for dSno is to facilitate Activin signaling during optic lobe formation in the third instar larval brain. Here via a series of assays we report that another developmental role for dSno is to spatially restrict Wg signaling in third instar larval wing disks. To date TGF-β-independent functions for mammalian SnoN have been identified in myoblasts [30] and cerebellar neurons [31] in culture and Ski has been found to associate with β-catenin in human melanoma cells [32] but no Sno family member has been reported to impact Wg signaling during development in any species.

### Genetic evidence for the mechanism for dSno antagonism of Wg signaling

*dSno* mutant clones cell-autonomously express the Wg target gene Ac on the wing blade but have no effect on normal Ac expression suggesting a role for dSno in antagonizing ectopic Wg signaling. Analysis of Wg expression in these clones eliminated the possibility that loss of *dSno* affects the transcription or translation of Wg. Coexpression experiments ruled out a role for dSno in Notch signaling and as a partner for Brk in wing disks.

**Figure 8:**
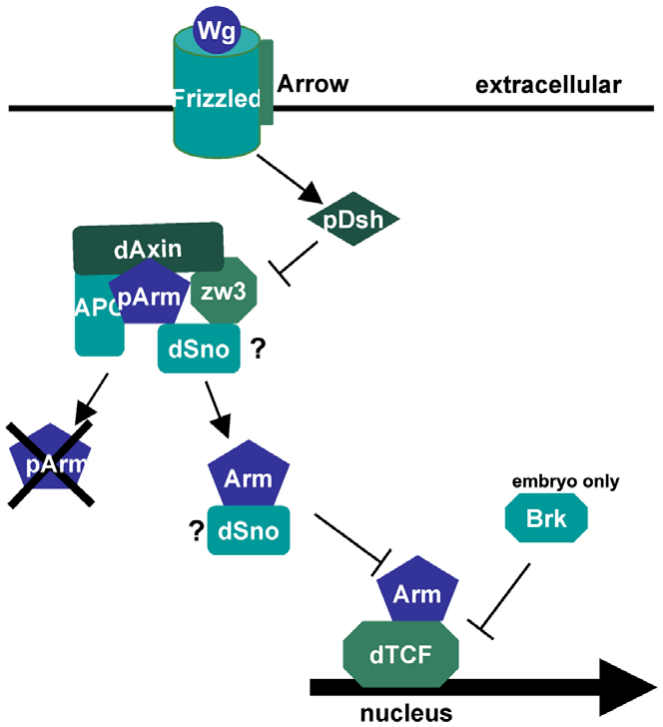
Potential placement of dSno in the Wingless pathway. A model depicting the Wg signal transduction pathway is shown. In the pathway Dsh, Arm and dTCF act positively while Zw3, Axin, APC and dSno act negatively. Based on epistasis data, we propose two possible locations were dSno may be acting (indicated as question marks) within the Wg pathway. The first possibility is that dSno cooperates with the other antagonistic proteins Zw3, dAxin and APC (representing dAPC1 and dAPC2). The second possibility is that dSno blocks Arm activity at a point subsequent to the destruction complex and prevents it from regulating Wg target genes. The embryonic Wg antagonist Brinker does not inhibit Wg functions during wing development.

Coexpression epistasis assays were able to specify where dSno might be acting in the Wg pathway (summarized schematically in Figure 8). dSno rescues the lethality and bristle phenotype of overexpression of Dsh placing dSno in the Wg pathway at the level of Dsh or below. Extending this result, dSno fully rescues the lethality and ectopic bristle phenotypes of Zw3-DN. This transgene generates modest overstimulation of Wg signaling (33.3% versus 99.16% lethality for overexpression of Dsh) because the kinase mutation reduces its ability to phosphorylate Arm and to amplify a Wg signal by phosphorylating Arrow [33]. These results suggest that dSno acts at or below the negative role for Zw3 whose loss generates the observed phenotypes.

Consistent with this placement, dSno overexpression enhanced the margin bristle phenotype of Zw3-CA. This transgene only affects the antagonistic role of Zw3 and generates reduced Wg activity. This is because of the sequential nature of Zw3 activity in Wg signaling - if Zw3 cannot be released from a complex with Arm by phosphorylation of its inhibiting serine then Zw3 will be unable to phosphorylate Arrow to amplify Wg signals. The enhancement data also suggest dSno acts at or below the antagonistic role for Zw3 in Wg signaling whose constitutive activity generates the observed phenotypes. The enhancing effect of UAS.dSno expression on UAS.dAxin and UAS.dAxinΔRGS phenotypes is similar to that seen with UAS.Zw3-CA suggesting that dSno acts at or below dAxin.

The fully epistatic effects of dSno on Dsh, Zw3 and dAxin were not reiterated in studies with Arm^S10^. Here mixed epistasis results were obtained. dSno coexpression resulted in the partial rescue of lethality and the suppression of ectopic dorsal bristles but did not influence the presence of ectopic ventral bristles or the anterior margin phenotype. The mixed results suggest that dSno antagonism of Wg signal transduction occurs at the level of Arm or above.

Taken together, the results suggest that dSno acts at or below the antagonistic cytoplasmic complex containing Zw3 and dAxin and at or above Arm to restrict ectopic Wg signaling. Thus, dSno is likely distinct from other Wg inhibitors such as Naked cuticle (inhibition of Dsh) [34] or Eyelid (transcriptional repression of target genes) [35]. Further, as Zw3 directly interacts with Arm in the cytoplasmic complex that includes dAPC1/dAPC2 and dAxin each of these proteins are candidates for targets of dSno binding in Wg signaling.

Lastly, although we have not yet identified the biochemical basis for dSno - Wg pathway interactions we have continued our analysis of dSno - Smad complex formation. Our previous data showed that dSno is capable of binding to Medea and dSmad2 but not to Mad [8] as reported for mammalian SnoN [3]. We analyzed a series of dSno point mutants to test the hypothesis that the same residues are employed in dSno - Medea binding as are involved in SnoN - Smad4 complex formation. The analysis demonstrated that dSno interactions with Medea are accomplished via the homologous amino acids in flies and mammals (Figure S7). This finding raises two intriguing possibilities: that antagonism of Wg signaling by Sno proteins is conserved in mammals and that dSno may provide a bridge for crosstalk between TGF-β and Wnt signaling.

In summary, we report an unexpected developmental role for dSno as a tissue-specific protein in Wg signaling with modest antagonistic activity under normal conditions in wing development but that effectively blocks extraneous Wg signals. Genetic evidence suggests the hypothesis that dSno antagonizes Wg signaling via a protein-protein interaction mechanism in cooperation with members of the cytoplasmic Arm destruction complex. A cytoplasmic role as an antagonist of Wg signaling and a nuclear role in facilitating TGF-β signaling may underlie the observation that the relative abundance of cytoplasmic versus nuclear SnoN is a prognostic indicator in a subset of tumors [36]. Perhaps the solution to the question of whether Sno proteins are oncogenes or tumor suppressor genes is that, depending upon the tissue, they may be both.

## Materials and Methods

### Drosophila strains

Fly stocks are as described: Achaete-lacZ [37], Arm-lacZ [38], *dSno^sh1402^*, *dSno^Ex17B^*, *dSno^Ex4B^* and UAS.dSno [8], *dSno^174^* [11], *dSno^GS-C517T^* [10], MS1096.Gal4 [21], [22], P{neoFRT}40A [39], P{FRT(w[hs])}101 [40], UAS.Arm^S10^ [25], UAS.Axin and UAS.Axin ΔRGS [27], UAS.Brk [29], UAS.Dsh [23], UAS.lacZ [41], UAS.MamN [42], UAS.CA-Notch [43], UAS.DN-Notch [44], UAS.Zw3-DN and UAS.Zw3-CA [26].

### Genetic analyses

Mutant clones: Recombinant chromosomes carrying *dSno^Ex17B^* FRT40A or *dSno^sh1402^* FRT40A were generated by standard methods. *dSno^Ex17B^* or *dSno^sh1402^* clones in wing disks were marked with Arm-lacZ FRT40A [45]. Briefly, larvae heterozygous for second chromosomes containing *dSno^EX17B^* FRT40A and Arm-lacZ FRT40A were heat shocked to express FLP recombinase from an X chromosome insertion at 72–96 hours after egg deposition to generate numerous small clones. Arm-lacZ is ubiquitously expressed [38]. All cells descendent from an initially heterozygous cell rendered homozygous for *dSno^EX17B^* or *dSno^sh1402^* by recombination were unambiguously visualized by the absence of lacZ.

Gal4-UAS studies: MS1096.Gal4 is an insertion in the X-linked gene Beadex that has a hemizygous wing phenotype in males but is fully recessive in females [21], [22]. Thus, in every mating the MS1096.Gal4 chromosome was contributed by a female parent and only female offspring that would be heterozygous for MS1096.Gal4 were considered as candidates for experimental individuals. Discrimination between experimental female adults and non-experimental siblings (an important internal control group) was accomplished with visible markers on balancer chromosomes. Female larvae were identified during imaginal disk dissection as described [46]. All full wing images are shown at the same magnification to aide comparison between genotypes. High magnification images are sized to maximize visibility of the anterior margin bristles and are not to scale.

Control experiments: Tests for Gal4 titration in strains with multiple UAS transgenes were conducted by substituting UAS.lacZ for UAS.dSno as described [47].

Statistics: To quantitate any observed lethality UAS transgenes were placed over a marked balancer in the parental strain and then the percent of expected adult progeny inheriting the transgene was calculated with reference to the number of siblings inheriting the balancer chromosome.

### Immunohistochemistry

Antibody labeling: The analysis of wing disks followed [48]. The following polyclonal antibodies were utilized: anti-lacZ (rabbit, Organon Teknika) and anti-Senseless (guinea pig) [49]. The following mouse monoclonal antibodies were obtained from the Developmental Studies Hybridoma Bank: anti-Achaete, anti-Cut (2B10), anti-Delta (C594.9B), anti-lacZ (JIE7), anti-Notch-Intracellular domain (C17.9C6) and anti-Wg (4D4). A mouse monoclonal antibody against Zw3 (2G2C5) [50] was a gift from Marc Bourouis. The following secondary antibodies were utilized: Alexa Fluor 488- and 633-conjugated goat anti-rabbit, anti-guinea pig and anti-mouse (Molecular Probes).

Microscopy: Images were collected on a Leica SP2 confocal microscope as a series of optical sections encompassing both cell layers of the wing disk. Each section was 0.18 µm thick and taken every 2.0 µm. Images displayed are compilations ranging in size from 14 to 24 optical sections. Images are sized to maximize visibility of the antibody labeling and are not to scale.

## Supporting Information

Text S1 Accompanying text, procedures and references for Supplemental Figures.
(0.07 MB PDF)

Figure S1 Comparative genomic analysis of four *dSno* mutants. (A) The coordinate line represents 105649base pairs from polytene region 28D3 (Genbank AE014134.5 - Release 5.22 sequence of *D. melanogaster* chromosome 2L - Dec 2009). Five resident genes (dSno is composed of two predictions CG7233 and CG7093) sized roughly to scale with their transcriptional orientations are shown above the line. The splicing pattern of the longest transcript encoding dSnoN (the longest protein isoform) is also shown. The nucleotide locations of the transcription start site and the initiator methionine for isoform are indicated below the coordinate line. (B) *dSno^sh1402^* contains a precise insertion of a P{lacW} transposon and a precise deletion (not shown) of a 297-class transposable element that is present in the 2L reference sequence. *dSno^sh1402^* is missing one of the three known dSno promoters and acts as a modest hypomorph. This data was previously shown in [1] as part of Fig. 5 but it has been updated here to match the base pair numbers of Release 5.22. (C) *dSno^Ex17B^* is a deletion of 5023 bp when compared to *dSno^sh1402^* that deletes the three known dSno promoters, the adjacent CG7231 and the 5′ end of CG7228. *dSno^Ex17B^* acts as a strong hypomorph. (D) *dSno^Ex4B^* is a deletion of 20849 bp when compared to *dSno^sh1402^* that deletes all dSno promoters, CG7233 (corresponding to the dSnoI protein isoform), CG7231, CG7224, CG7228 but not CG7224. *dSno^Ex4B^* is a protein null. (E) As reported in [2], *dSno^174^* is a deletion of 9518 bp when compared to *dSno^sh1402^*. The deletion begins at amino acid 57 removing the remaining 276 amino acids of CG7233 and the splice acceptor creating essentially a protein null.
(4.87 MB TIF)

Figure S2 *dSno* transcription is significantly reduced in *dSno^Ex17B^* embryos and similar to Wg expression in the ventral epidermis.Embryos in lateral view. (A) Stage 17 wild type embryo hybridized with a dSnoI riboprobe displaying strong *dSno* expression in the brain and ventral cord. Additional expression in segmentally reiterated stripes in the ventral epidermis is indicated with red arrowheads. (B) Stage 15 homozygous *dSno^Ex17B^* embryo with weak staining in the brain and ventral cord. (C) Left side - Stage 17 transheteroygous *dSno^Ex17B^*/*dSno^Ex4B^* mutant embryo with weak staining in the brain and ventral cord that is significantly less than in wild type. Right side - Stage 17 embryo heterozygous for a *dSno* excision allele balanced over CyOP{wg-lacZ}. This sibling embryo is a control for embryo genotype and the staining reaction. (D)Stage 17 wild type embryo revealing that *dpp* RNA is present in many tissues but not in the ventral epidermis (red arrowheads). (E) Stage 16 wild type embryo with Wg protein expression visible in the ventral epidermis that corresponds to regions that will generate naked cuticle.
(4.45 MB TIF)

Figure S3 *dSno* is expressed in the optic lobe and *dSno* mutant optic lobes display reduced cell proliferation. A) In a wild type third instar larval optic lobe, a dSnoI riboprobe reveals prominent expression in the presumptive lamina plexus and medulla neuropil (black arrowhead). B-C) Optic lobes stained with antibodies to Brdu (green) and Elav (red). An arrowhead indicates the inner proliferation zone of the medulla neuropil. B) Wild type lobe has a well-defined inner proliferation zone containing numerous cells in S-phase. C) Transheteroygous *dSno^174^*/*dSno^Ex4B^* mutant lobe with an ill-defined inner proliferation zone containing a reduced number of cells in S phase. This result is consistent with previous optic lobe data showing that *dSno^sh1402^*/*dSno^Ex4B^* mutants have reduced numbers of cells in M phase [1].
(1.70 MB TIF)

Figure S4 *dSno^Ex17B^* wing clones and loss of function genotypes phenocopy clones of the Wg pathway antagonist *zw3*. (A, A') Wild type wing. (B, B') Wings with unmarked clones of *dSno^Ex17B^* display up to eight individual ectopic margin bristles in the distal region of the anterior compartment of the wing blade (arrowheads). (C, C') Wings with unmarked clones of *zw3^M11^* display numerous ectopic margin bristles, individual bristles as well as clusters of bristles, throughout the wing blade due to loss of Zw3 antagonism for Wg signaling. (D, D') Wings with unmarked clones of the Wg transcription factor *arm* (*arm^4^*) are missing margin bristles due to the loss of Wg signaling. (E, E') Wings of *dSno^174^* homozygous escapers display up to ten individual ectopic margin bristles in distal and medial regions of the anterior compartment. (F, F') Wings of *dSno^EX4B^*/*dSno^GS-C517T^* transheterozygous escapers display up to five ectopic margin bristles in the distal region of the anterior compartment.
(7.60 MB TIF)

Figure S5 *dSno* loss of function genotypes display ectopic sensilla, a phenotype not associated with the loss of Dpp signaling. (A) Wild type wing. (B) High magnification view of three campaniform sensilla on the dorsal surface of longitudinal vein3 (L3) in a wild type wing (arrowheads). (C) *dSno^174^* homozygous escaper with five campaniform sensilla on L3 (four are shown - arrowheads). (D) Wing from Fig. S4C with unmarked clones of *zw3^M11^* has four campaniform sensilla on L3 (arrowheads). (E) Scabrous.Gal4;UAS.lacZ pupal disk stained with anti-lacZ. Note prominent expression in the L3 primordia (arrowhead). (F) Sca.Gal4; UAS.dSno wing with most of L3 missing due to antagonism of Dpp signal transduction and is also missing two of the L3 sensilla (the remaining one is indicated with an arrowhead). (G) Sca.Gal4; UAS.Mad-RNAi wing with all of L3 missing due to loss of Dpp signal transduction but all L3 sensilla are present (arrowheads). (H) Sca.Gal4; UAS.Dsh wing with ectopic bristles on L3 due to ectopic Wg signaling. (I) Sca.Gal4; UAS.Dsh, UAS.dSno rescued wing with one remaining ectopic bristle due to dSno antagonism of ectopic Wg signaling but also with most of L3 missing due to dSno antagonism of Dpp signal transduction.
(7.62 MB TIF)

Figure S6 *dSno* mutant embryos do not have altered Wg expression but they have ectopic expression of a Wg target gene in the ventral epidermis. (A) Wild type embryo. Each hemisegment (2 are shown) of the ventral cuticle contains six rows of denticles in a trapezoidal pattern pointing to the anterior and a region of equal size with no denticles. (B) *wg^en1^* homozygous loss of function embryo. All ventral cells have denticles. (C) *wg^Gla^* heterozygous gain of function embryo. Tissue-specific and non-lethal *wg* overexpression prevents any ventral cells from producing denticles. Note that the loss of denticles is not fatal - this embryo would eventually become an adult with a Glazed eye phenotype resulting from a second round of Wg overexpression in eye disks. (D) *dSno^sh1402^* homozygous loss of function embryo. This embryo with no denticles is similar to a *wg^Gla1^* (gain of function) embryo. Note that these denticle-less embryos would eventually hatch but they do not survive past the pupal stage due to other defects. (E) Stage 13 *dSno^sh1402^* heterozygous embryo labeled to reveal the expression of segmentally reiterated stripes of Wg protein (green) and Wg RNA (red). An enhancer trap in wg present on the CyO balancer chromosome expresses lacZ and the embryo was stained with an antibody to lacZ. (F) Stage 13 homozygous *dSno^sh1402^* embryo (no lacZ staining due to the absence of the balancer chromosome) with wild type expression of Wg protein. (G) Stage 14 wild type embryo labeled to display segmentally reiterated stripes of En expression (each En stripe is located immediately posterior to a Wg stripe and En is a target of Wg). The one to two cells wide stripe of En expression is visible in the inset. (H) Stage 14 homozygous *dSno^sh1402^* embryo with expanded En expression in each stripe. The width of each stripe of En staining is expanded to three to four cells (inset).
(5.05 MB TIF)

Figure S7 dSno - Medea binding is conserved between mammals and flies. (A) Deletion of amino acids 1–69 or 1–108 from dSno did not affect Medea interaction. The T280Y mutation in dSno decreased the intensity of Medea interaction. (B) The W283E mutation in dSno abolishes Medea interaction as does the dSno double mutant T280Y and H271A. (C) Deletion of amino acids 1–108 of dSno decreases recruitment of dSmad2 to dSno - Medea complexes: compare the amount of dSmad2 in lane 4 with lane 6. Reduction in dSno - Medea binding by the T280Y mutation also leads to reduced binding of dSmad2: compare lane 4 with lane 8. (D) Analysis of a deletion series covering the first 108 amino acids of dSno reveals that only the first 13 amino acids are required for dSmad2 recruitment to Medea - dSno complexes. (E) Schematic of dSno mutants with an amino acid scale bar and domains as indicated: blue is Medea interaction, purple is a coiled-coil and gray is a region of significant identity between predicted Sno proteins from 12 Drosophila species (D. Wotton; unpublished observations). Also shown are effects on dSno - Medea binding or Medea - dSno complex recruitment of dSmad2: +  =  interaction, -  =  no interaction, +/−  =  weak interaction and nd  =  not determined.
(9.69 MB TIF)
