# Supplementary material for: The Sno Oncogene Antagonizes Wingless Signaling during Wing Development in Drosophila
Source: PLoS One. 2010 Jul 16;5(7):e11619. doi: 10.1371/journal.pone.0011619 (PMC2905394; doi:10.1371/journal.pone.0011619)
Supplement: Text S1 — Accompanying text, procedures and references for Supplemental Figures. (0.07 MB PDF) [file pone.0011619.s001.pdf]

Text for Supplemental Figures 1 - 3

Sequence, expression and complementation studies of *dSno*^{Ex17B}, *dSno*^{Ex4B} and *dSno*^{174}

Since our initial paper on dSno [1], we sequenced the breakpoints associated with our excision alleles (Figure S1). Sequencing revealed that *dSno*^{Ex17B} is a deletion of 5023bp when compared to the parental *dSno*^{sh1402} allele or 11940bp when compared to the *D. melanogaster* chromosome arm 2L reference sequence due to the presence of the 6917bp 297 retrotransposon in the reference sequence but not *dSno*^{sh1402}. *dSno*^{Ex17B} deletes the three dSno promoters we identified previously as well as the neighboring gene CG7231 and part of the next gene CG7228. CG7231 is a gene of unknown function and we found that *dSno*^{Ex17B} fully complemented four homozygous viable P insertions in this gene - P{EPgy2}EY11884, P{EP}2510, P{SUPor-P}KG04307, P{wHy}DG01605. CG7228 was identified in an cell culture RNAi screen in blood cells as required to permit mycobacterial infection [3]. It has 13 known viable transposon insertions and none have any reported organismal mutant phenotype. Thus, we conclude that the mutant phenotype of *dSno*^{Ex17B} mutants is attributable to the loss of dSno. This conclusion is supported by evidence that *dSno*^{Ex17B} mutants can be rescued with UAS.dSno [1]. We examined *dSno* RNA expression in *dSno*^{Ex17B} homozygous and transheterozygous mutant embryos (Figure S2) and found a low level of wild type expression Taken together the sequence and expression data suggest that while the dSno open reading frame is present *dSno*^{Ex17B} acts as a strong hypomorphic allele.

Sequencing revealed that *dSno*^{Ex4B} is a deletion of 20849bp when compared to *dSno*^{sh1402} or 27766bp with regard to the *D. melanogaster* chromosome arm 2L reference sequence. The deletion removes the exon at the amino terminus of all dSno proteins that contains the Smad4-binding amino acids. It also removes the neighboring genes CG7231, CG7228 and CG7227. We conclude that *dSno*^{Ex4B} is a protein null but that its mutant phenotype likely includes a contribution from the loss of CG7227 (for which no mutants are currently available). This supposition is supported by data showing that homozygous *dSno*^{Ex4B} mutants cannot be rescued with UAS.dSno [1].

As reported in [2], *dSno*^{174} is a deletion of 9518bp when compared to *dSno*^{sh1402}. The deletion begins at amino acid 57 removing the remaining 276 amino acids of CG7233 and the splice acceptor creating essentially a dSno protein null. An examination of *dSno*^{174} homozygous flies (generously provided by Siegfried Roth) showed that they are extremely weak and survive for just two to three days. A stage of a lethality test (n=300) revealed that homozygous *dSno*^{174} individuals who do not reach adulthood die during pupal stages consistent with our lethality data from *dSno*^{sh1402} homozygous and *dSno*^{sh1402} / *dSno*^{Ex17B} genotypes [1]. Complementation tests between *dSno*^{174} and *dSno*^{sh1402} or *dSno*^{Ex17B} also generated 50% pupal lethality in transheterozygous individuals. Importantly, *dSno*^{174} failed to complement *dSno*^{Ex4B}. As *dSno*^{Ex4B} and *dSno*^{174} are essentially protein null alleles it appears that in our laboratory the homozygous deletion of dSno coding sequences is lethal while in other laboratories it can be viable.

RNA in situ experiments revealed that dSno is expressed in the medulla neuropil of the optic lobes of third instar larvae (Figure S3). Examination of *dSno*^{174} / *dSno*^{Ex4B} transheterozygous third instar larval optic lobes showed that they display reduced cell proliferation in the medulla neuropil, the same defect in Activin signaling that we reported for *dSno*^{sh1402} / *dSno*^{Ex4B} transheterozygous larvae [1]. Thus, one possible explanation for the discrepancy in *dSno* mutant lethality is that environmental factors in our laboratory such as food content or ambient humidity reduce the viability of transheterozygous *dSno* deletion mutants to zero. Support for this explanation is found in reports of independently generated *dSno* homozygous deletion mutants from two other laboratories that are viable at only 30% of expected [4,5]. Taken together, our complementation studies suggest that all of the reported *dSno* mutants are allelic, that *dSno* plays a role in facilitating Activin signaling in optic lobe development and that the extent of viability for *dSno* homozygous deletions varies between laboratories due to environmental factors.

Text for Supplemental Figures 4-5

Loss of *dSno* generates phenotypes associated with ectopic Wg signaling

In a wild type wing (Figure S4), formation of the wing margin and its associated bristles depends upon Wg signaling. Loss of the Wg antagonist *zw3* in unmarked mutant clones results in the activation of Wg target genes and the formation of ectopic bristles outside the margin on the wing blade [6]. This phenotype can also be generated by eliciting ectopic Wg signaling via overexpression of the Dishevelled signal transducer [7]. Alternatively, loss of the Wg effector *arm* in unmarked mutant clones at the margin prevents normal bristle formation [8]. Unmarked clones of cells homozygous for either of the excision mutants *dSno*^{Ex17B} or *dSno*^{Ex4B} (not shown) and the P-element insertion *dSno*^{sh1402} (not shown) display ectopic bristles in distal regions of the anterior compartment the wing blade.

To insure this phenotype corresponded to the loss of *dSno* and not another mutation on the chromosome when examined wings from *dSno* mutants generated in other labs. First we examined wings from *dSno*^{174} homozygous escapers [5]. *dSno*^{174} is an open reading frame deletion generated by excision of the P element in *dSno*^{sh1402}, a strategy similar to the one that created *dSno*^{Ex17B} and *dSno*^{Ex4B}. *dSno*^{174} escapers display ectopic margin bristles on the wing blade in distal regions of the anterior compartment.

We then examined wings of *dSno*$^{GS\text{-}C517T}$ homozygous escapers [4]. *dSno*$^{GS\text{-}C517T}$ is an EMS induced mutation resulting in a premature stop codon 173 amino acids downstream of the dSno initiator methionine. It was generated in the enhancer piracy line *P{GS}*^{18054} where the P element is inserted upstream of the dSno coding region. The homozygous escaper rate for *dSno*$^{GS\text{-}C517T}$ is greater, in our hands, than that of *dSno*^{174} and thus *dSno*$^{GS\text{-}C517T}$ does not appear to be a *dSno* null allele. The extent to which the *dSno* gene is compromised in this line is difficult to characterize for two reasons. First, the P element remains in place potentially impacting *dSno* transcription. Second, there is an in-frame methionine at position 246 of the dSno open reading frame that would result in a potentially functional protein containing the Medea-interacting amino acids should it be employed as cryptic translation initiator. Nevertheless, examining escapers from this line is important to eliminate the possibility of background effects as it was generated on a totally distinct chromosome. We did not observe any ectopic bristles in the wings of *dSno*$^{GS\text{-}C517T}$

homozygous escapers. However, when this allele is placed in trans to *dSno*^{Ex4B} (a deletion of the dSno open reading frame) escaper wings display ectopic bristles at the distal end of the anterior compartment of their wings.

The presence of ectopic bristles in three dSno mutant genotypes examined led us to more closely analyze *dSno*^{174} homozygous escaper wings for other evidence of ectopic Wg signaling. We noted that these wings display ectopic campaniform sensilla (Figure S5). In a wild type wing there three campaniform sensilla on the dorsal surface of longitudinal vein3 (L3) and two on the dorsal surface of L1. *dSno*^{174} homozygous escapers have four or five campaniform sensilla on L3 often an extra sensilla on L1. Sensilla develop from sensory organ precursors just like anterior margin bristles and their development are also regulated by Wg.

We confirmed that this phenotype is due to loss of Wg antagonism in *dSno* mutants in loss of function and gain of function experiments. First, an examination of wings with unmarked clones of *zw3*^{M11} revealed that they also display ectopic sensilla on L3. This finding directly implicates the loss of Wg antagonism in this phenotype. Second, overexpression of dSno with Scabrous.Gal4 (Sca.Gal4 has prominent expression in the L3 primordia of pupal wings) resulted in loss of the L3 vein due to Dpp antagonism and the loss of sensilla on L3 due to Wg antagonism. We confirmed that the loss of L3 sensilla in the genotype is not due to loss of Dpp signaling in experiments with Mad-RNAi. Expression of Mad-RNAi eliminated the L3 vein completely but had no effect on the L3 sensilla.

Text for Supplemental Figure 6

***dSno* mutant embryos display ectopic expression of a Wg target in the ventral epidermis**

The wing disk results led us to examine the possibility that there are embryonic ectodermal tissues in which *dSno* might antagonize Wg signaling. In a careful reexamination of our *dSno* RNA in situ hybridization data [1] we noted something we missed earlier in our focus on *dSno's* prominent CNS expression. In fact ectoderm-wide expression of *dSno* at stage 14 refines by stage 16 not only to the CNS but also to narrow, segmentally repeated stripes in the

ventral epidermis. This aspect of *dSno* expression is distinct from any feature of *dpp* expression at this stage but appears similar to Wg expression in the same tissue (Figure S2).

The embryonic ventral epidermis is composed of reiterated segments that contain twelve rows of cells (oriented perpendicular to the anterior/posterior axis) - six rows that secrete smooth cuticle followed by six rows that secrete protrusions called denticles in a trapezoidal pattern pointing to the anterior (Figure S4). Cells choose to secrete smooth cuticle or denticles according to positional information supplied, in part, by Engrailed (En) and Wg [9,10]. During stages 10-12, secreted Wg signals permit cells to secrete smooth cuticle by suppressing, indirectly, the expression of En. En is expressed just posterior to Wg and an En expressing cell secretes the first denticle row. In a *wg* loss of function mutant En expression in the ventral epidermis is expanded (visible at stage 12 and beyond) and therefore all twelve rows of cells secrete denticles [11]. Alternatively in a *wg* gain of function mutant with tissue-specific overexpression in the ventral epidermis (*wg*^{Gla}) [12], En is uniformly suppressed and all twelve rows of cells are smooth.

To determine if loss of *dSno* had any phenotypic consequences we examined cuticles from *dSno*^{sh1402} embryos and *dSno*^{sh1402} / *dSno*^{Ex17B} transheterozygous embryos. We found that these embryos display extensive smooth cuticle - a phenotype that mimics a *wg* overexpressing embryo. To be certain the *dSno* mutant phenotype was due to effects on Wg signal transduction and not due to effects on Wg expression we examined Wg and En expression in *dSno* mutant embryos. Wg expression was normal in dSno^{sh402} or *dSno*^{sh402} / *dSno*^{EX17B} embryos at stage 13. Thus, the cuticle phenotype of *dSno* mutants is not due loss of Wg itself.

Careful examination of the expression of the Wg target gene En revealed that in dSno mutants En expression is one-two cell diameters wider than in wild type embryos, a result similar to that seen in a *wg* gain of function mutant embryo [13]. As in the wing, in a *dSno* mutant there is both normal and ectopic expression of a Wg target gene in the ventral epidermis.

Text for Supplemental Figure 7

dSno and Medea interact via a mechanism conserved in flies and mammals

The initial set of experiments showed that deletion of the first 108 amino acids from dSno does not affect its ability to bind Medea (Figure S5). We also tested the point mutation T280Y that affects an amino acid in dSno that is homologous to one of three amino acids in human Sno family proteins that bind Smad4 [14,15]. This mutation decreased the intensity of the dSno - Medea interaction. The W283E mutation in dSno affecting a second amino acid homologous to a Smad4 binding amino acid abolishes Medea interaction as does the dSno double mutant T280Y/H271A. H271 in dSno is homologous to the third Smad4 binding amino acid in human Sno family members. The data shows that dSno - Medea binding requires the homologous residues in dSno that are important for SnoN - Smad4 binding in mammals.

A second group of experiments was designed to study dSno - Medea - dSmad2 complexes, an important mechanism by which dSno modulates Medea activity. We found that deletion of amino acids 1 - 108 of dSno decreases recruitment of dSmad2 to dSno - Medea complexes and that reduction in dSno - Medea binding by the T280Y mutation also leads to reduced incorporation of dSmad2 into the complexes. A deletion series within the first 108 amino acids of dSno reveals that only the first 13 amino acids are required for dSmad2 recruitment to Medea - dSno complexes. Overall the biochemical studies suggest that dSno - Medea binding requires the homologous residues in dSno important for SnoN - Smad4 binding in mammals. Alternatively, recruitment of dSmad2 to dSno - Medea complexes is dissimilar to mammalian SnoN where the Smad2/3 binding amino acids are numbers 90-94.

Supplemental Procedures

Excision breakpoint sequencing (Figure S1)

A series of nineteen PCR primer sets, each just over 1kb apart, were designed across the region from CG7233 to CG7224 (base pairs 7999743 - 7970749 within the chromosome arm 2L reference sequence; Genbank AE014134.5). The break points of $dSno^{Ex4B}$ and $dSno^{Ex17B}$ were first roughly determined by the successful amplification of a PCR product from sequences both upstream and downstream of the deleted regions. Then the forward primer of the upstream

primer pair and the reverse primer of the downstream primer pair were employed together to amplify a junction fragment that spans the two break points in each deletion. For *dSno*^{Ex4B} the forward primer was 5'tagcccctcattttcacagc3' and the reverse primer was 5'cgccactcgtcgatagatag3'. For *dSno*^{Ex17B} the forward primer was 5'aactggcggagatgcttg3' and the reverse primer was 5'cagggcttatgacgaatatgg3'. PCR fragments were then cloned into the pCR2.1 TOPO vector (Invitrogen) and sequenced using the forward and reverse PCR primers that were used to amplify the fragments. The sequences were then analyzed by BLAST against the 2L reference sequence to find the exact break points of each deletion allele.

RNA in situ hybridization (Figures S2 and S3)

Embryos: The *dSno*^{Ex17B} and *dSno*^{Ex4B} mutations were each balanced over CyO- P{*wg*^{en1}-lacZ}. The absence of lacZ RNA expression was employed to identify *dSno*^{Ex17B} homozygous mutant embryos or transheterozygous *dSno*^{Ex17B} / *dSno*^{Ex4B} mutant embryos in egg collections. Double labeling of embryos by RNA in situ hybridization with *lacZ* and *dSnoI* riboprobes or with a *dpp* riboprobe was conducted as described [16]. Optic lobes: RNA in situ hybridization with a *dSnoI* riboprobe was conducted as described [17].

Antibody staining (Figures S2, S3, S5 and S6)

Embryos: Antibody labeling of wild type and *dSno* mutant embryos (identified via the absence of lacZ staining from CyO-P{*wg*^{en1}-lacZ}) was conducted as described [18]. The following antibodies were utilized: α-lacZ (rabbit, Organon Teknika), α-Engrailed (4D9; DSHB), and α-Wg (4D4; DSHB), Alexa Fluor 488- and 633-conjugated goat α-rabbit and α-mouse (Molecular Probes). Optic lobes: Brdu labeling followed by antibody staining was conducted as described [17]. The following antibodies were utilized: α-Brdu (G3G4; DSHB) and α-Elav (7E8A10; DSHB**),** Alexa Fluor 488- and 633-conjugated goat α-rat and α-mouse (Molecular Probes). Pupal wings at 18-20 hours after pupariation: α-lacZ (rabbit, Organon Teknika) staining was conducted as described [1].

Drosophila genetics (Figures S3, S4, S5 and S6)

Optic lobes: The *dSno*^{174} and *dSno*^{Ex4B} mutations were balanced over CyO-GFP. The absence of GFP expression was employed to identify transheterozygous mutant larvae for dissection and staining. Wing clones: *arm*^{4} and *zw3*^{M11} unmarked wing clones were generated with FRT101 while *dSno*^{Ex4B}, *dSno*^{Ex17B} and *dSno*^{sh1402} unmarked wing clones were generated with FRT 40A following [19]. *arm*^{4} (*arm*^{YD35}) and *zw3*^{M11} are as described [8,20]. Gal4-UAS wings: Sca.Gal4 was from Bloomington (stock #6479), UAS.MadRNAi was a kind gift from Mike O'Connor. Embryos: *wg*^{en1} - a *P* element insertion creating a lacZ-expressing loss of function allele on the CyO balancer chromosome [11], *wg*^{Gla1} - a *roo* element insertion causing dominant non-lethal phenotypes due to tissue-specific overexpression in eye disks [21] and the ventral epidermis [12] are as described. Cuticles were prepared as described [22] from a stock of *dSno*^{sh1402} / CyO-P{*wg*^{en1}-lacZ} and from a cross of *dSno*^{sh1402} / CyO-P{*wg*^{en1}-lacZ}with *dSno*^{Ex17B} / CyO-P{*wg*^{en1}-lacZ} flies. *dSno* mutant cuticles were identified by elimination of heterozygous (wild type) and homozygous balancer (*wg*^{en1}) cuticles. For the *dSno*^{sh1402} / CyO-*wg*^{en1} stock (n = 157) we scored 77.7% of cuticles as wild type, 10.2% as *wg*^{en1} and 12.1% as *dSno* mutants.

Biochemistry (Figure S7)

dSmad2 and Medea wild type cDNAs were T7-tagged and a wild type dSno cDNA was Flag-tagged as described [1]. dSno deletion constructs and point mutants were created by PCR from the wild type cDNA via standard methods and expressed from pCMV5 with an amino-terminal Flag-tag. Cell culture, transfection methods, co-immunoprecipitation and western blotting were as described [1].

Supplemental References

1. Takaesu N, Hyman-Walsh C, Ye Y, Wisotzkey R, Stinchfield M, O'Connor M, Wotton D,

Newfeld S (2006) dSno facilitates Baboon signaling in the Drosophila brain by switching the affinity of Medea away from Mad and toward dSmad2. Genetics 174: 1299-1313.

2. Shravage B, Altmann G, Technau M, Roth S (2007) The role of Dpp and its inhibitors during eggshell patterning in Drosophila. Development 134: 2261-2271.

3. Philips J, Rubin E, Perrimon N (2005) Drosophila RNAi screen reveals CD36 family member required for mycobacterial infection. Science 309: 1251-1253.

4. Ramel M, Emery C, Foulger R, Goberdhan D, van den Heuvel M, et al. (2007) Drosophila SnoN modulates growth and patterning by antagonizing TGF-□ signals. Mech Dev 124: 304-317.

5. Barrio R, López-Varea A, Casado M, de Celis J (2007) Characterization of *dSnoN* and its relationship to Decapentaplegic signaling in Drosophila. Dev Biol 306: 66-81.

6. Blair S (1992) *shaggy* (*zeste-white 3*) and the formation of supernumerary bristle precursors in the developing wing blade of Drosophila. Dev Biol 152: 263-278.

7. Penton A, Wodarz A, Nusse R (2002) A mutational analysis of *dishevelled* in Drosophila defines novel domains in the Dishevelled protein as well as novel suppressing alleles of *axin*. Genetics 161: 747-762.

8. Peifer M, Wieschaus E (1990) The segment polarity gene *armadillo* encodes a functionally modular protein that is the Drosophila homolog of human plakoglobin. Cell 63: 1167-1176.

9. Gritzan U, Hatini V, DiNardo S (1999) Mutual antagonism between signals secreted by adjacent Wg and En cells leads to specification of complementary regions of the Drosophila parasegment. Development 126: 4107-4115.

10. Alexandre C, Lecourtois M, Vincent J-P (1999) Wingless and Hedgehog pattern Drosophila denticle belts by regulating the production of short-range signals. Development 126: 5689-5698.

11. Kassis J, Noll E, VanSickle P, Odenwald W, Perrimon N (1992) Altering the insertional specificity of Drosophila transposable element. Proc Natl Acad Sci USA 89: 1919-1923.

12. Takaesu N, Bulanin D, Johnson A, Orenic T, Newfeld S (2008) A combinatorial enhancer recognized by Mad, TCF and Brinker first activates then represses *dpp* expression in the

posterior spiracles of Drosophila. Dev Biol 313: 829-843.

13. Kremer S, Erdeniz N, Peterson-Nedry W, Swanson E, Wehrli M (2010) In vivo analysis in Drosophila reveals differential requirements of contact residues in Axin for interactions with GSK3β or β-catenin. Dev Biol 337: 110-123

14. Stroschein S, Wang W, Zhou S, Zhou Q, Luo K (1999) Negative feedback regulation of TGF-β signaling by the SnoN oncoprotein. Science 286: 771-774.

15. Wu J, Krawitz A, Chai J, Li W, Zhang F, Luo K, Shi Y (2002) Structural mechanism of Smad4 recognition by Ski, insights repression of TGF-β. Cell 111: 357-367.

16. Takaesu N, Johnson A, Sultani O, Newfeld S (2002) Combinatorial signaling by an unconventional Wg pathway and the Dpp pathway requires Nejire (CBP) to regulate *dpp* expression in posterior tracheal branches. Dev Biol 247: 225-236.

17. Brummel T, Abdollah S, Haerry T, Shimell M, Merriam J, Raftery L, Wrana J, O'Connor M (1999) The Drosophila Activin receptor Baboon signals through dSmad2 and controls cell proliferation but not patterning during larval development. Genes Dev 13: 98-111.

18. Johnson A, Burnett L, Sellin J, Paululat A, Newfeld S (2007) Defective Dpp signaling results in heart overgrowth and reduced cardiac output in Drosophila. Genetics 176: 1609-1624.

19. Wilder E, Perrimon N (1995) Dual functions of *wingless* in the Drosophila leg imaginal disc. Development 121: 477-488.

20. Siegfried E, Chou T, Perrimon, N (1992) *wingless* signaling acts through *zeste-white 3*, the Drosophila homolog of GSK-3, to regulate *engrailed* and establish cell fate. Cell 71: 1167-1179.

21. Brunner E, Brunner D, Fu W, Hafen E, Basler K (1999) The dominant mutation *Glazed* is a gain-of-function allele of *wingless* that, similar to loss of APC, interferes with normal eye development. Dev Biol 206: 178-188.

22. Wharton K, Ray R, Gelbart W (1993) A gradient of Decapentaplegic is necessary for the specification of dorsal pattern elements in the Drosophila embryo. Development 117: 807-822.
