# Supplementary figures and images for: The Sno Oncogene Antagonizes Wingless Signaling during Wing Development in Drosophila
Source: PLoS One. 2010 Jul 16;5(7):e11619. doi: 10.1371/journal.pone.0011619 (PMC2905394; doi:10.1371/journal.pone.0011619)

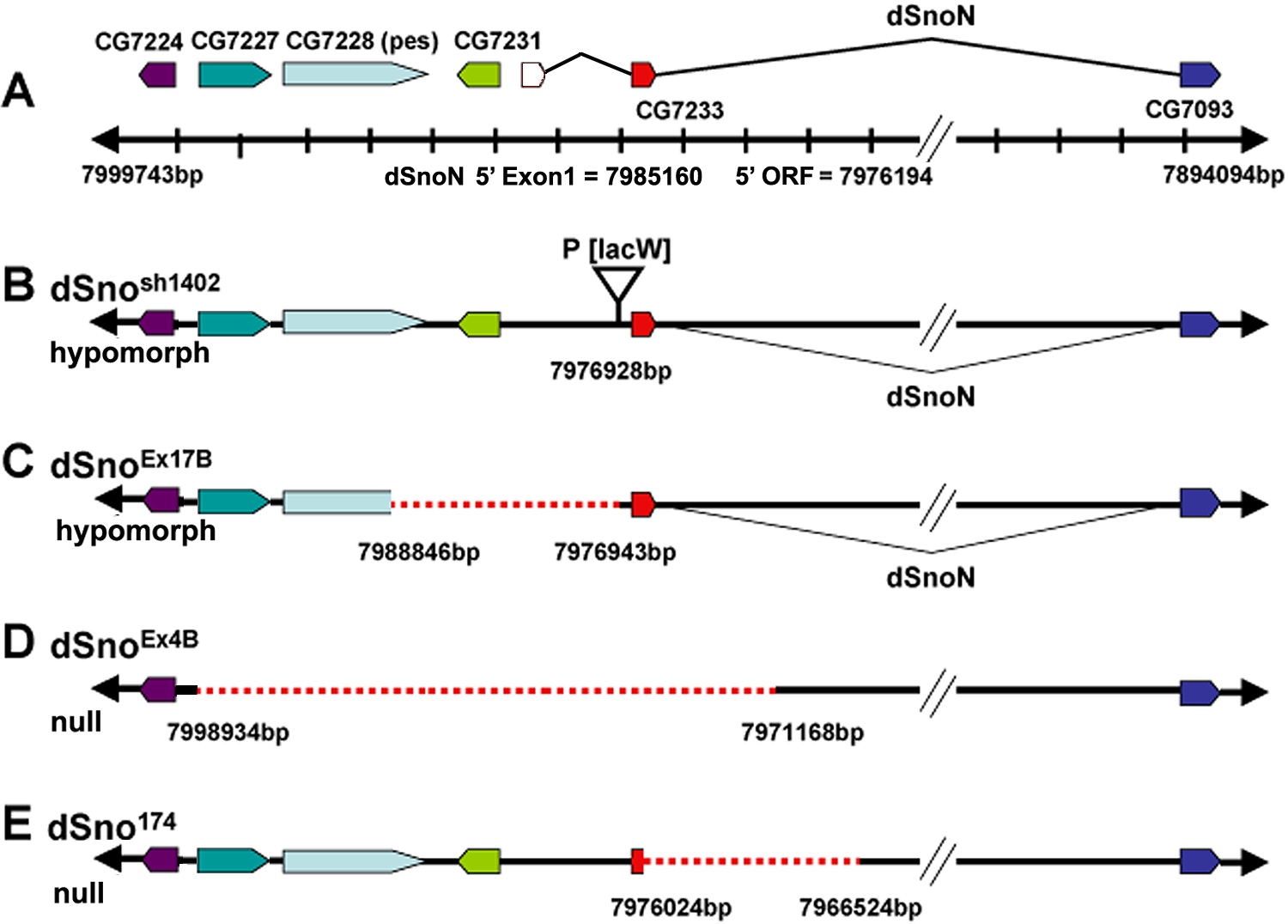

Supplement: Figure S1 — Comparative genomic analysis of four dSno mutants. (A) The coordinate line represents 105649base pairs from polytene region 28D3 (Genbank AE014134.5 - Release 5.22 sequence of D. melanogaster chromosome 2L - Dec 2009). Five resident genes (dSno is composed of two predictions CG7233 and CG7093) sized roughly to scale with their transcriptional orientations are shown above the line. The splicing pattern of the longest transcript encoding dSnoN (the longest protein isoform) is also shown. The nucleotide locations of the transcription start site and the initiator methionine for isoform are indicated below the coordinate line. (B) dSnosh1402 contains a precise insertion of a P{lacW} transposon and a precise deletion (not shown) of a 297-class transposable element that is present in the 2L reference sequence. dSnosh1402 is missing one of the three known dSno promoters and acts as a modest hypomorph. This data was previously shown in [1] as part of Fig. 5 but it has been updated here to match the base pair numbers of Release 5.22. (C) dSnoEx17B is a deletion of 5023 bp when compared to dSnosh1402 that deletes the three known dSno promoters, the adjacent CG7231 and the 5′ end of CG7228. dSnoEx17B acts as a strong hypomorph. (D) dSnoEx4B is a deletion of 20849 bp when compared to dSnosh1402 that deletes all dSno promoters, CG7233 (corresponding to the dSnoI protein isoform), CG7231, CG7224, CG7228 but not CG7224. dSnoEx4B is a protein null. (E) As reported in [2], dSno174 is a deletion of 9518 bp when compared to dSnosh1402. The deletion begins at amino acid 57 removing the remaining 276 amino acids of CG7233 and the splice acceptor creating essentially a protein null. (4.87 MB TIF) [file pone.0011619.s002.tif]

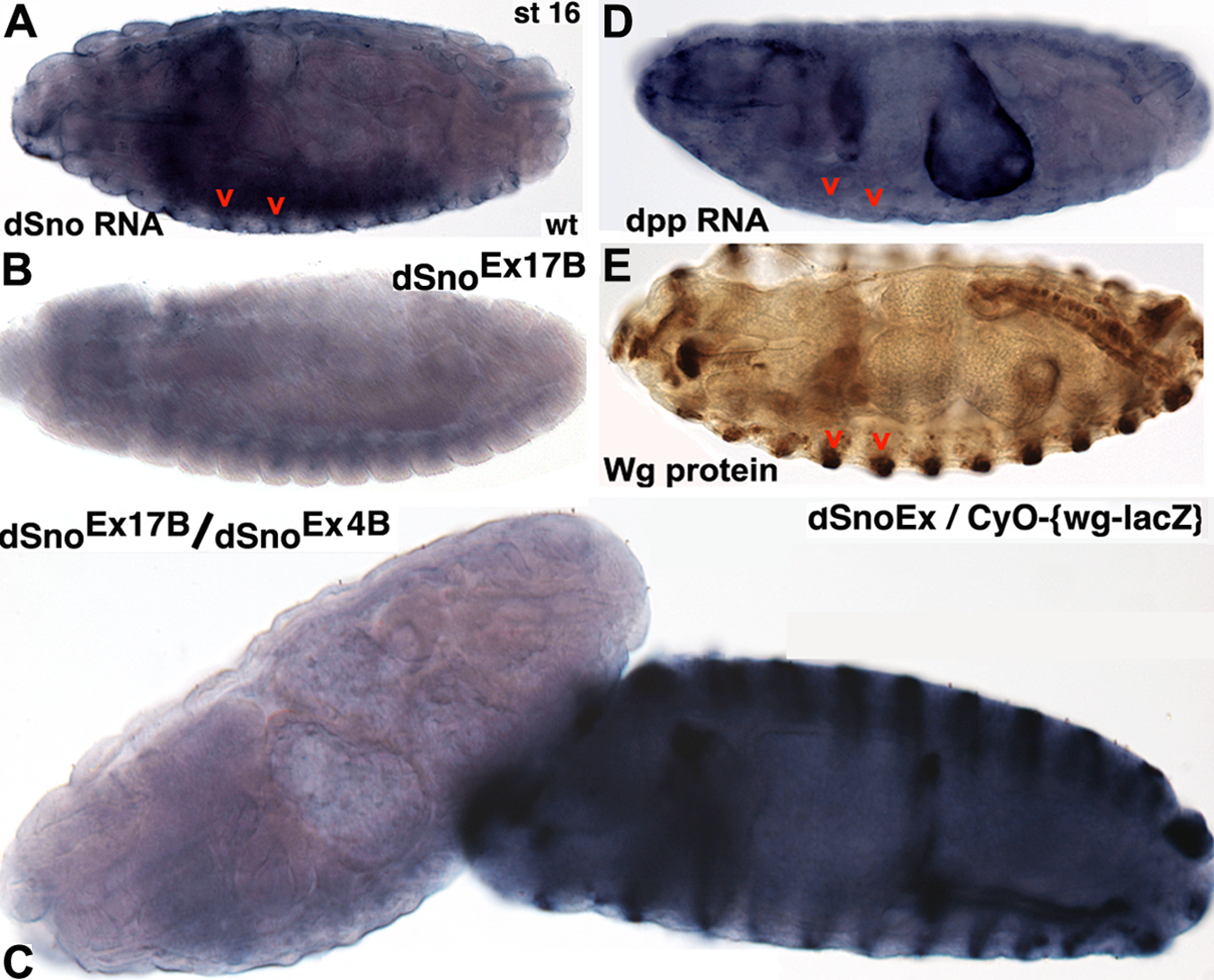

Supplement: Figure S2 — dSno transcription is significantly reduced in dSnoEx17B embryos and similar to Wg expression in the ventral epidermis.Embryos in lateral view. (A) Stage 17 wild type embryo hybridized with a dSnoI riboprobe displaying strong dSno expression in the brain and ventral cord. Additional expression in segmentally reiterated stripes in the ventral epidermis is indicated with red arrowheads. (B) Stage 15 homozygous dSnoEx17B embryo with weak staining in the brain and ventral cord. (C) Left side - Stage 17 transheteroygous dSnoEx17B/dSnoEx4B mutant embryo with weak staining in the brain and ventral cord that is significantly less than in wild type. Right side - Stage 17 embryo heterozygous for a dSno excision allele balanced over CyOP{wg-lacZ}. This sibling embryo is a control for embryo genotype and the staining reaction. (D)Stage 17 wild type embryo revealing that dpp RNA is present in many tissues but not in the ventral epidermis (red arrowheads). (E) Stage 16 wild type embryo with Wg protein expression visible in the ventral epidermis that corresponds to regions that will generate naked cuticle. (4.45 MB TIF) [file pone.0011619.s003.tif]

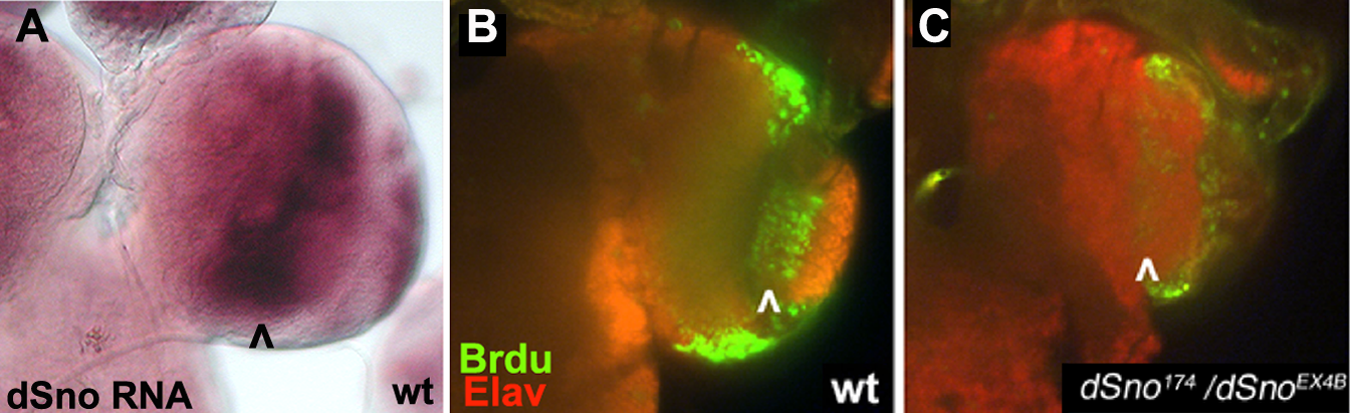

Supplement: Figure S3 — dSno is expressed in the optic lobe and dSno mutant optic lobes display reduced cell proliferation. A) In a wild type third instar larval optic lobe, a dSnoI riboprobe reveals prominent expression in the presumptive lamina plexus and medulla neuropil (black arrowhead). B-C) Optic lobes stained with antibodies to Brdu (green) and Elav (red). An arrowhead indicates the inner proliferation zone of the medulla neuropil. B) Wild type lobe has a well-defined inner proliferation zone containing numerous cells in S-phase. C) Transheteroygous dSno174/dSnoEx4B mutant lobe with an ill-defined inner proliferation zone containing a reduced number of cells in S phase. This result is consistent with previous optic lobe data showing that dSnosh1402/dSnoEx4B mutants have reduced numbers of cells in M phase [1]. (1.70 MB TIF) [file pone.0011619.s004.tif]

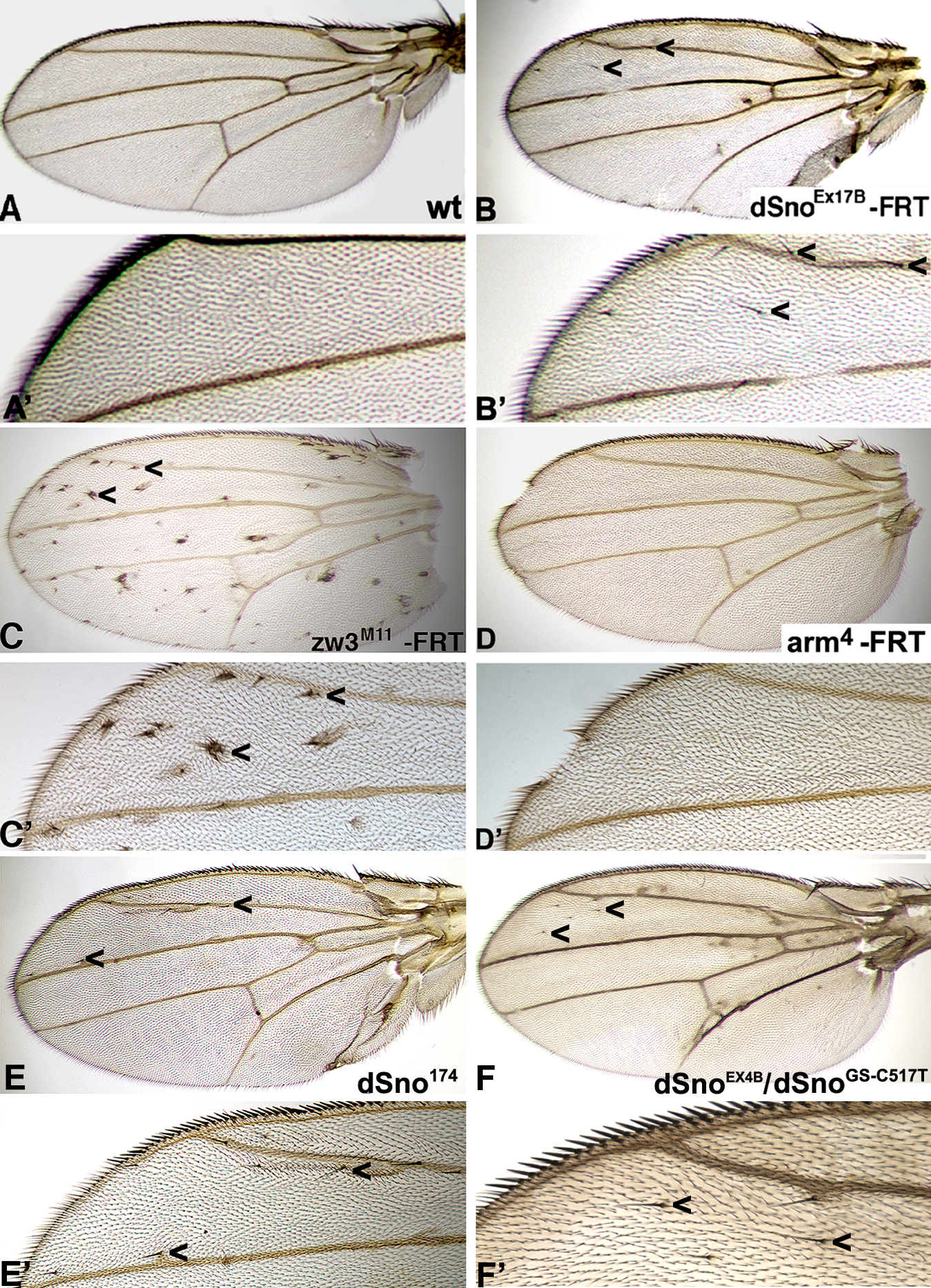

Supplement: Figure S4 — dSnoEx17B wing clones and loss of function genotypes phenocopy clones of the Wg pathway antagonist zw3. (A, A') Wild type wing. (B, B') Wings with unmarked clones of dSnoEx17B display up to eight individual ectopic margin bristles in the distal region of the anterior compartment of the wing blade (arrowheads). (C, C') Wings with unmarked clones of zw3M11 display numerous ectopic margin bristles, individual bristles as well as clusters of bristles, throughout the wing blade due to loss of Zw3 antagonism for Wg signaling. (D, D') Wings with unmarked clones of the Wg transcription factor arm (arm4) are missing margin bristles due to the loss of Wg signaling. (E, E') Wings of dSno174 homozygous escapers display up to ten individual ectopic margin bristles in distal and medial regions of the anterior compartment. (F, F') Wings of dSnoEX4B/dSnoGS-C517T transheterozygous escapers display up to five ectopic margin bristles in the distal region of the anterior compartment. (7.60 MB TIF) [file pone.0011619.s005.tif]

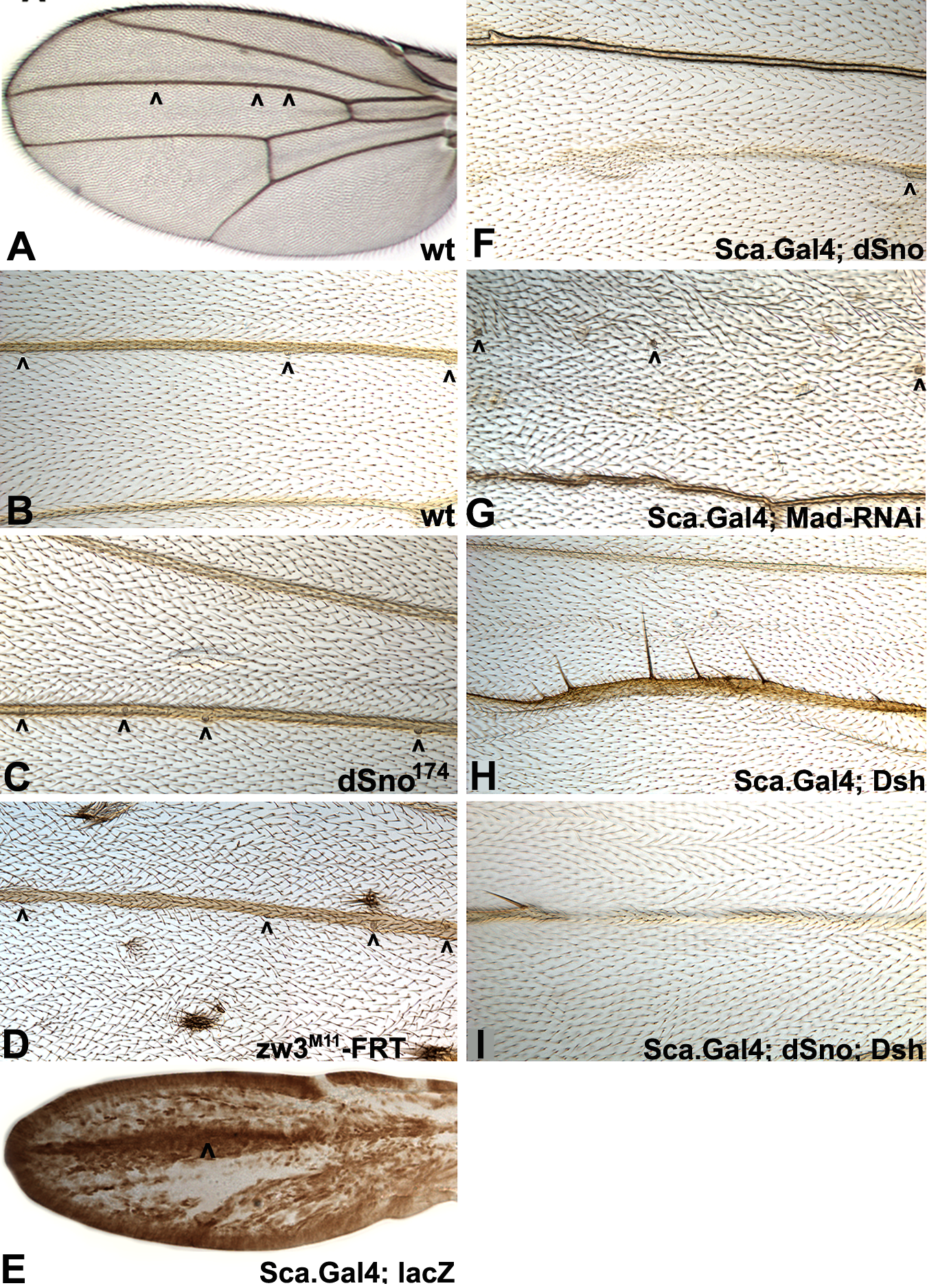

Supplement: Figure S5 — dSno loss of function genotypes display ectopic sensilla, a phenotype not associated with the loss of Dpp signaling. (A) Wild type wing. (B) High magnification view of three campaniform sensilla on the dorsal surface of longitudinal vein3 (L3) in a wild type wing (arrowheads). (C) dSno174 homozygous escaper with five campaniform sensilla on L3 (four are shown - arrowheads). (D) Wing from Fig. S4C with unmarked clones of zw3M11 has four campaniform sensilla on L3 (arrowheads). (E) Scabrous.Gal4;UAS.lacZ pupal disk stained with anti-lacZ. Note prominent expression in the L3 primordia (arrowhead). (F) Sca.Gal4; UAS.dSno wing with most of L3 missing due to antagonism of Dpp signal transduction and is also missing two of the L3 sensilla (the remaining one is indicated with an arrowhead). (G) Sca.Gal4; UAS.Mad-RNAi wing with all of L3 missing due to loss of Dpp signal transduction but all L3 sensilla are present (arrowheads). (H) Sca.Gal4; UAS.Dsh wing with ectopic bristles on L3 due to ectopic Wg signaling. (I) Sca.Gal4; UAS.Dsh, UAS.dSno rescued wing with one remaining ectopic bristle due to dSno antagonism of ectopic Wg signaling but also with most of L3 missing due to dSno antagonism of Dpp signal transduction. (7.62 MB TIF) [file pone.0011619.s006.tif]

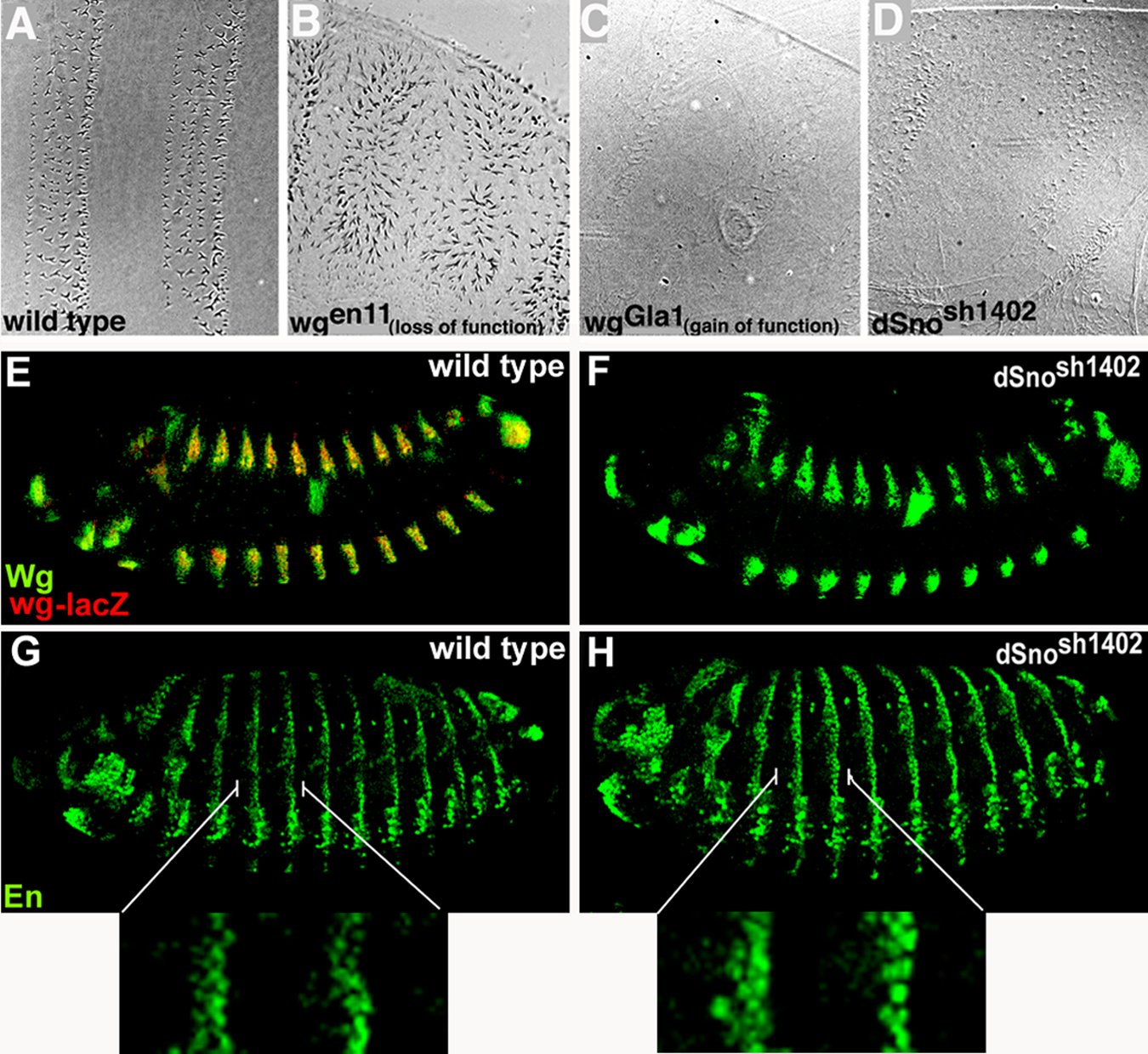

Supplement: Figure S6 — dSno mutant embryos do not have altered Wg expression but they have ectopic expression of a Wg target gene in the ventral epidermis. (A) Wild type embryo. Each hemisegment (2 are shown) of the ventral cuticle contains six rows of denticles in a trapezoidal pattern pointing to the anterior and a region of equal size with no denticles. (B) wgen1 homozygous loss of function embryo. All ventral cells have denticles. (C) wgGla heterozygous gain of function embryo. Tissue-specific and non-lethal wg overexpression prevents any ventral cells from producing denticles. Note that the loss of denticles is not fatal - this embryo would eventually become an adult with a Glazed eye phenotype resulting from a second round of Wg overexpression in eye disks. (D) dSnosh1402 homozygous loss of function embryo. This embryo with no denticles is similar to a wgGla1 (gain of function) embryo. Note that these denticle-less embryos would eventually hatch but they do not survive past the pupal stage due to other defects. (E) Stage 13 dSnosh1402 heterozygous embryo labeled to reveal the expression of segmentally reiterated stripes of Wg protein (green) and Wg RNA (red). An enhancer trap in wg present on the CyO balancer chromosome expresses lacZ and the embryo was stained with an antibody to lacZ. (F) Stage 13 homozygous dSnosh1402 embryo (no lacZ staining due to the absence of the balancer chromosome) with wild type expression of Wg protein. (G) Stage 14 wild type embryo labeled to display segmentally reiterated stripes of En expression (each En stripe is located immediately posterior to a Wg stripe and En is a target of Wg). The one to two cells wide stripe of En expression is visible in the inset. (H) Stage 14 homozygous dSnosh1402 embryo with expanded En expression in each stripe. The width of each stripe of En staining is expanded to three to four cells (inset). (5.05 MB TIF) [file pone.0011619.s007.tif]

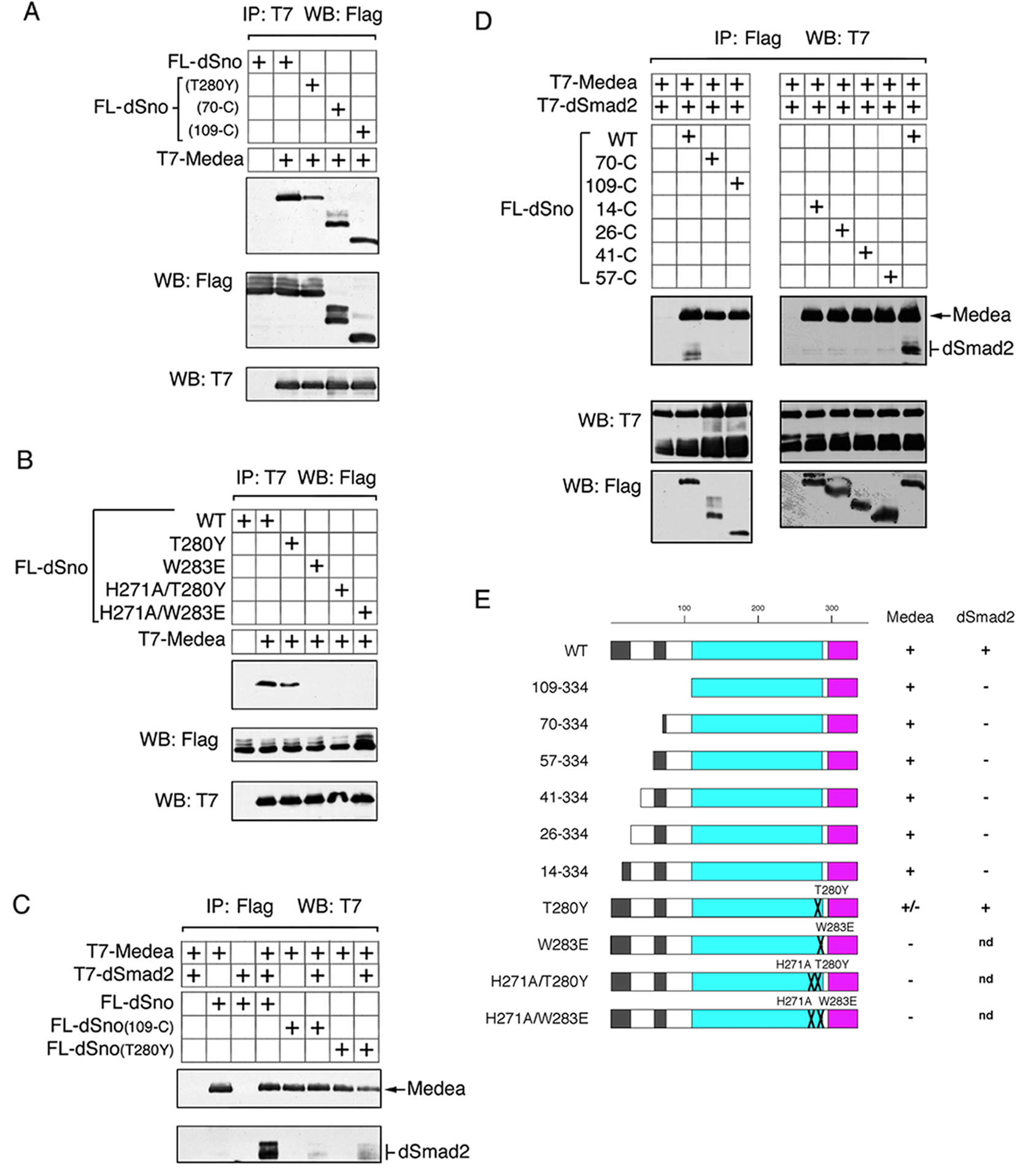

Supplement: Figure S7 — dSno - Medea binding is conserved between mammals and flies. (A) Deletion of amino acids 1–69 or 1–108 from dSno did not affect Medea interaction. The T280Y mutation in dSno decreased the intensity of Medea interaction. (B) The W283E mutation in dSno abolishes Medea interaction as does the dSno double mutant T280Y and H271A. (C) Deletion of amino acids 1–108 of dSno decreases recruitment of dSmad2 to dSno - Medea complexes: compare the amount of dSmad2 in lane 4 with lane 6. Reduction in dSno - Medea binding by the T280Y mutation also leads to reduced binding of dSmad2: compare lane 4 with lane 8. (D) Analysis of a deletion series covering the first 108 amino acids of dSno reveals that only the first 13 amino acids are required for dSmad2 recruitment to Medea - dSno complexes. (E) Schematic of dSno mutants with an amino acid scale bar and domains as indicated: blue is Medea interaction, purple is a coiled-coil and gray is a region of significant identity between predicted Sno proteins from 12 Drosophila species (D. Wotton; unpublished observations). Also shown are effects on dSno - Medea binding or Medea - dSno complex recruitment of dSmad2: + = interaction, - = no interaction, +/− = weak interaction and nd = not determined. (9.69 MB TIF) [file pone.0011619.s008.tif]
